# Cost-effectiveness of post-landing latent tuberculosis infection control strategies in new migrants to Canada

**DOI:** 10.1371/journal.pone.0186778

**Published:** 2017-10-30

**Authors:** Jonathon R. Campbell, James C. Johnston, Mohsen Sadatsafavi, Victoria J. Cook, R. Kevin Elwood, Fawziah Marra

**Affiliations:** 1 Faculty of Pharmaceutical Sciences, University of British Columbia, Vancouver, British Columbia, Canada; 2 Division of Respiratory Medicine, Faculty of Medicine, University of British Columbia, Vancouver, Canada; 3 British Columbia Centre for Disease Control, Vancouver, British Columbia, Canada; Fundació Institut d’Investigació en Ciències de la Salut Germans Trias i Pujol, Universitat Autònoma de Barcelona, SPAIN

## Abstract

**Background:**

The majority of tuberculosis in migrants to Canada occurs due to reactivation of latent TB infection. Risk of tuberculosis in those with latent tuberculosis infection can be significantly reduced with treatment. Presently, only 2.4% of new migrants are flagged for post-landing surveillance, which may include latent tuberculosis infection screening; no other migrants receive routine latent tuberculosis infection screening. To aid in reducing the tuberculosis burden in new migrants to Canada, we determined the cost-effectiveness of using different latent tuberculosis infection interventions in migrants under post-arrival surveillance and in all new migrants.

**Methods:**

A discrete event simulation model was developed that focused on a Canadian permanent resident cohort after arrival in Canada, utilizing a ten-year time horizon, healthcare system perspective, and 1.5% discount rate. Latent tuberculosis infection interventions were evaluated in the population under surveillance (N = 6100) and the total cohort (N = 260,600). In all evaluations, six different screening and treatment combinations were compared to the base case of tuberculin skin test screening followed by isoniazid treatment only in the population under surveillance. Quality adjusted life years, incident tuberculosis cases, and costs were recorded for each intervention and incremental cost-effectiveness ratios were calculated in relation to the base case.

**Results:**

In the population under surveillance (N = 6100), using an interferon-gamma release assay followed by rifampin was dominant compared to the base case, preventing 4.90 cases of tuberculosis, a 4.9% reduction, adding 4.0 quality adjusted life years, and saving $353,013 over the ensuing ten-years. Latent tuberculosis infection screening in the total population (N = 260,600) was not cost-effective when compared to the base case, however could potentially prevent 21.8% of incident tuberculosis cases.

**Conclusions:**

Screening new migrants under surveillance with an interferon-gamma release assay and treating with rifampin is cost saving, but will not significantly impact TB incidence. Universal latent tuberculosis infection screening and treatment is cost-prohibitive. Research into using risk factors to target screening post-landing may provide alternate solutions.

## Background

In Canada, over two-thirds of all active tuberculosis (TB) cases occur in migrants [1–3]. Current pre-immigration TB screening protocols are mandatory for permanent residents and select temporary residents; screening consists of a medical history, chest x-ray (CXR) and sputum tests to rule out active TB. Migrants diagnosed with active TB must complete an adequate course of therapy before migrating to Canada. Meanwhile, those with a medical history or CXR suggestive of prior TB are flagged for post-landing surveillance—approximately 2% [4]. The follow-up system is passive, with adherence to post-landing surveillance reported to be between 60 to 70% [1,4].

The post-landing surveillance system is successful in identifying people at risk for active TB after arrival; in Ontario one-third of all active TB in the first two years post-migration occurred in those flagged for surveillance. However, genotypic studies estimate that approximately 85% of all TB cases in migrants are due to reactivation of latent TB infection (LTBI) acquired prior to migration [5–7]. In those with LTBI, approximately 5–10% will progress to active TB over their lifetime, but effective treatment can reduce risk of progression by over 90% [8]. Despite this, it is unknown how many migrants flagged for surveillance are screened for LTBI and for the remaining 98% not flagged, there is no routine LTBI screening protocol, leaving a large group of migrants at risk for active TB and a missed opportunity for TB prevention [4]. Implementation of a LTBI screening system, however, would have to overcome inefficiencies in the LTBI cascade of care. In this context, the cascade of care consists of placing a screening test, evaluating the result, performing a medical evaluation, initiating and completing treatment. At the present, high rates of dropout during screening and treatment result in <20% of those who may benefit from treatment actually completing it [9].

Evidence-based screening and treatment recommendations in new migrants need to support TB elimination efforts in Canada. Implementation of pre- or post-landing LTBI screening protocols have been suggested [1,10–12], but no system or policy is in place to execute any of these possible solutions. In this study we aim to provide evidence surrounding possible implementation of post-landing LTBI screening. We developed a model to determine the cost and prevalence of LTBI and imported active TB in recent migrants. These estimates were then applied to view the impact LTBI screening post-landing would have on TB incidence in subgroups of a cohort of new migrants to Canada.

## Methods

### Study population

The population studied in the model was the 2014 cohort of new permanent residents to Canada, which consists of 260,600 new permanent residents, of which 6100 were flagged for post-landing medical surveillance. The cohort was characterized by post-landing surveillance flag, derived from Ontario data [4], age and TB incidence in country of origin, derived from Immigration, Refugees and Citizenship Canada [13], and Bacillus Calmette-Guérin (BCG) vaccination status, based on countries with a current national vaccination policy for all, which was derived from the BCG World Atlas [14] and adjusted based on 36-year average reported immunization rates [15]. To determine the prevalence of LTBI and imported (non-preventable) TB in this population, an optimization scheme was developed. Two-year TB incidence rates in new permanent resident cohorts to Ontario between 2002 and 2011 [4], stratified by TB incidence in country of origin (low <30 cases per 100,000 population, moderate 30–99 cases, high 100–199 cases, and very high ≥200 cases) and surveillance flag were used as optimization targets. Several assumptions were made. Firstly, we assumed 85% of TB cases in those not flagged for surveillance were due to reactivation of LTBI [5–7] and that the rate of reactivation was constant over time [16]. Second, those flagged for surveillance had a reactivation risk 3.9 times higher than those not flagged for surveillance, selected based on TB risks from a long-term study in Britain [17]. Finally, it was assumed that LTBI prevalence in those under surveillance was higher than those who were not. To optimize to the targets, the baseline average reactivation rate was varied between 0.8 and 1.6 reactivation TB cases per 1000 person-years [18–21] and the proportion of TB cases in those flagged for surveillance that were imported was varied between 55% and 85% [16,22]. After optimizing these parameters to our targets, these estimates were applied proportionally to the demographic profile of the 2014 permanent resident cohort. Results of the optimization and more detailed optimization rationale and methods are outlined in S1 Text, S1 and S2 Tables.

### Discrete event simulation model

A discrete event simulation (DES) model was developed in Simio and run using Simio Replication Runner (Version 8.146.14121, Simio LLC, Sewickley, PA). DES was chosen as it allowed for variable cycle times, simultaneous events to occur to each migrant, and enabled creating many parameters describing each patient (a Markov model would have too many states to accommodate the same level of granularity). The model’s time horizon was 10 years from arrival to Canada to minimize extrapolation from optimization targets. The model took a healthcare system perspective and used a discount rate of 1.5% for costs and outcomes as recommended by the Canadian Agency for Drugs and Technologies in Health [23]. The model’s main outcomes were cost per quality adjusted life year (QALY) gained and cost per TB case prevented. A willingness-to-pay (WTP) threshold of $100,000 CAD per QALY [24–27] or $20,000 CAD per TB case prevented (i.e. approximately the average cost of managing one TB case) was used to determine if an intervention was cost-effective. Several assumptions were made in the model. Firstly, it was not possible to self-heal from LTBI without treatment. Secondly, multi-drug resistant TB was not considered due to extremely low incidence in Canada and difficulty in accurately costing cases. Finally, direct transmission between migrants in the cohort or to the general population was not modeled, rather we accounted indirectly for this through a certain proportion being remotely infected during the simulation.

**Fig 1:**
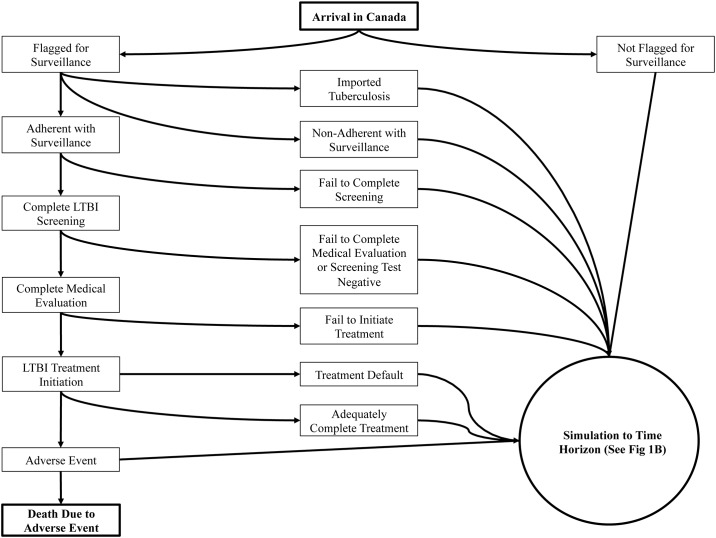
Model structure: Flow of new migrants through the simulation and the interventions investigated upon arrival in Canada. LTBI: Latent tuberculosis infection.

**Fig 2:**
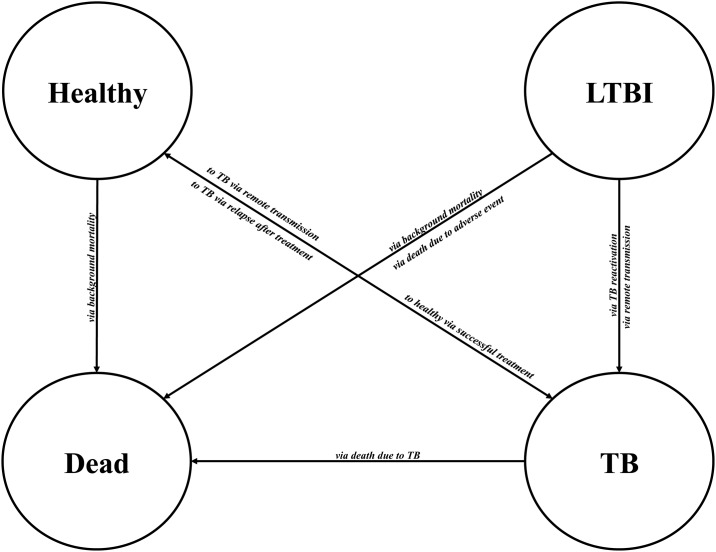
Possible events that may result in movement between health states after arrival in Canada. LTBI: Latent tuberculosis infection; TB: tuberculosis.

The model structure is outlined in Figs 1 and 2. Upon arrival, simulated migrants may import tuberculosis, be flagged for post-landing TB surveillance based on pre-immigration screening, or not be flagged for surveillance at all; those flagged for surveillance may or may not adhere. Migrants adhering are given an LTBI screening test and those completing the test that are positive are all referred and given a medical evaluation (to rule out active TB). Migrants completing the medical evaluation are offered LTBI treatment and should they initiate treatment are simulated to either default at some point during treatment, discontinue due to an adverse event, die due to fatal hepatotoxicity, or fully complete treatment. All migrants, regardless of their simulation pathway, are then simulated to the model’s time horizon, with an annual risk of developing TB or dying of background mortality. Upon development of TB, a chance of a remote TB case occurring was simulated to account for the proportion of TB cases in migrants not occurring due to reactivation (S1 Formula). Those who develop TB and complete treatment are at risk of experiencing TB relapse for the subsequent two years.

### Model characteristics

#### Input parameters

Published reports and expert opinion were used to estimate input parameters for the model. Where possible, systematic reviews were used to derive model estimates; in cases where this was not possible, estimates from the literature were used. Background mortality was derived from Canadian life tables [28]. LTBI diagnostic test sensitivity was derived from each test’s ability to detect prevalent TB (i.e. a surrogate measure) [29], while test specificity was derived in populations at very low risk of infection [30,31] and stratified by BCG vaccination status [14,15]. TB reactivation rate was carefully chosen from data from a variety of studies [7,18–21,32–35]. A rate of 1.1 per 1000 person years in individuals with LTBI was selected as it results in a cumulative incidence of TB of 5% over approximately 45 years and provides reasonable estimates of LTBI prevalence based on a meta-analysis of IGRA positivity in migrants [36]. Transition between all health states was modeled annually, except in the case of transition from adverse events or from TB to a subsequent health state, which had varying transition times. Table 1 lists all model estimates.

**Table 1:** Model parameters and analyses range.

Parameter	Estimate	Univariate Analysis Range	Range for PSA	Reference
**Costs**				
**Full INH Treatment**	$992	$804, $1179	Triangular, 804–1179	BCCDC, [37,38]
**Drug Costs**	$181			
**Nurse and Clinician Costs**	$741			
**Follow-up CXR**	$42			
**Routine Tests**	$28			
**Full RIF Treatment**	$575	$464, $686	Triangular, 464–686	BCCDC [37,38]
**Drug Costs**	$98			
**Nurse and Clinician Costs**	$421			
**Follow-up CXR**	$42			
**Routine Tests**	$14			
**Partial INH**	$462	N/A	Triangular, 174–804	BCCDC, [37,38]
**Partial RIF**	$319	N/A	Triangular, 178–464	BCCDC, [37,38]
**Complete TST**	$31	$24, $38	Triangular, 24–38	BCCDC, [37,38]
**TST Cost**	$11			
**Nurse Costs (Two Visits)**	$20			
**Incomplete TST**	$21	$17, $25	Triangular, 17–25	BCCDC, [37,38]
**IGRA**	$54	$31, $62	Triangular, 31–62	BCCDC, [37,38]
**Kit and Technician Cost**	$47			
**Nurse Costs**	$7			
**CXR**	$42	N/A	Triangular, 32–52	BCCDC, [37,38]
**Cost per X-Ray**	$35			
**Nurse Costs**	$7			
**Tuberculosis**	$20,532	$16,730, $24,334	Gamma(4.1064,5000)	Expert Opinion, [37,39]
**LTBI Adverse Event**	$732	$549, $916	Triangular, 549–916	[37]
**Hospitalization**	$6641	$5305, $9985	Triangular, 5305–9985	[40]
**Death**	$26,933	$13,079, $40,788	Triangular, 13,079–40,788	[41]
**QALYs**				
**LTBI**	0.81	0.75, 1.0	Beta(9.49,2.23)	[42–44]
**Healthy**	0.81	0.75, 1.0	Beta(7.85,1.84)	[42–44]
**Adverse Event Disutility**	0.2	0, 0.5	Triangular, ±25%	[37,40]
**TB**	0.69	0.55, 0.75	Beta(6.84,3.07)	[42–44]
**Hospitalization**	0.5	0.3, 0.7	Triangular, ±25%	[40]
**Dead**	0	-	-	-
**Screening Parameters**				
**TST Sensitivity**	0.782	0.50, 0.95	Beta(43,12)	[29,30]
**TST Specificity (No BCG)**	0.974	0.94, 1	Beta(770,21)	[30,31]
**TST Specificity (BCG)**	0.602	0.35, 0.87	Beta(239,158)	[30,31]
**IGRA Sensitivity**	0.889	0.81, 0.95	Beta(8,1)	[29,30]
**IGRA Specificity**	0.957	0.86, 1	Beta(900,40)	[30,31]
**IGRA Indeterminate**	0.06	0, 0.18	Beta(83,1286)	[31]
Complete TST*	0.72	0.72, 1.0	Beta(117.84,45.83)	[9,45]
**Complete Medical Evaluation**†	0.78	0.6, 1.0	Beta(46.12,13.01)	[9]
**Parameters for Population Under Surveillance**				
**Adherent with Surveillance**	0.605	0.7, 0.8	-	[4]
**LTBI Prevalence ≥200 cases**	0.3420	Varied with Reactivation Rate	Varied with Reactivation Rate	[4]
**LTBI Prevalence 100–199 cases**	0.3659	Varied with Reactivation Rate	Varied with Reactivation Rate	[4]
**LTBI Prevalence 30–99 cases**	0.1862	Varied with Reactivation Rate	Varied with Reactivation Rate	[4]
**LTBI Prevalence <30 cases**	0.0641	Varied with Reactivation Rate	Varied with Reactivation Rate	[4]
**Overall Imported TB Prevalence**	0.0054	-	-	[16]
**Parameters for Population Not Under Surveillance**				
**LTBI Prevalence ≥200 cases**	0.3162	Varied with Reactivation Rate	Varied with Reactivation Rate	[4]
**LTBI Prevalence 100–199 cases**	0.2016	Varied with Reactivation Rate	Varied with Reactivation Rate	[4]
**LTBI Prevalence 30–99 cases**	0.0902	Varied with Reactivation Rate	Varied with Reactivation Rate	[4]
**LTBI Prevalence <30 cases**	0.0159	Varied with Reactivation Rate	Varied with Reactivation Rate	[4]
**Treatment Parameters**				
**Initiate Therapy**^#^	0.938	0.5, 1	Beta(180.83,11.95)	[9,45]
**Complete INH**	0.616	0.5, 0.7	Beta(131.66,82.07)	[9]
**Complete RIF**	0.814	0.7, 0.9	Beta(76.85,17.56)	[9]
**Adverse Event INH**	0.060	0.04, 0.12	Beta(134,2095)	[46–50]
**Adverse Event RIF**	0.027	0.01, 0.07	Beta(56,2043)	[46–50]
**Hospitalization | AE**	0.01	0, 0.02	Beta(1,99)	[40]
**Death INH**	0.00000988	0, 0.0001	Beta(2,202495)	[51]
**LTBI Risk Reduction INH**	0.93	0.5, 1	Normal(-2.597,0.461)§	[8]
**LTBI Risk Reduction RIF**	0.8	0.5, 1	Normal(-1.609,0.500)§	[52,53]
**Partial Risk Reduction INH**	0.346	0, 0.69	Combination of Normal Distributionsǂ	[8,46–50]
**Partial Risk Reduction RIF**	0	0, 0.69	Normal(-0.693,0.300)§	[52,53]
**Adverse Event Duration**	7 days	3, 17	Gamma(0.7,10)	Expert Opinion, [40]
**TB Parameters**				
**Death from TB**	0.0476	0, 0.08	Beta(76,1523)	[1]
**Reactivation Rate**	0.0011	0.0009, 0.0013	Beta(90.92,82545.55)	[7,18–21,32–35]
**Risk Increase if Abnormal**	3.9	2.7, 5.5	Normal(1.36,0.15)§	[17]
**Extended Therapy**	0.124	0, 0.3	Beta(2.366,16.713)	Expert Opinion, [40]
**Relapse Rate**	0.0359	0.0274, 0.0462	Normal(-3.327,0.365)§	[54]
**Model Parameters**				
**Flagged for surveillance**	0.024	Optimization Parameter	Optimization Parameter	[4]
**BCG Vaccination (<30 cases)**	0.605	-	Beta(45137,29502)	[14]
**BCG Vaccination (≥30 cases)**	0.938	0.5, 1	Beta(180.83,11.95)	[9,45]
**BCG Vaccination Uptake**	0.616	0.5, 0.7	Beta(131.66,82.07)	[9]
**Discount Rate**	0.814	0.7, 0.9	Beta(76.85,17.56)	[9]
**Time Horizon**	0.060	0.04, 0.12	Beta(134,2095)	[46–50]

*Number imputed from 43.4% of migrants indicated for screening completing [9] (if 60.5% are adherent with surveillance, 72% must complete TST screening).

^†^Number imputed from 43.7 of 56 individuals referred for medical evaluation completing [9].

^#^This model assumes all who complete a medical evaluation and have no indication for active TB, are recommended treatment.

^§^The result is exponentiated (i.e. is a lognormal distribution).

^ǂ^Formula: 0.33*(Normal(-1.168,0.228))+0.374*(Normal(-0.381,0.169))+0.293*1

All costs are in 2016 CAD.

BCCDC: British Columbia Centre for Disease Control; QALY: Quality Adjusted Life Year; PSA: Probabilistic Sensitivity Analysis; INH: Isoniazid; RIF: Rifampin; TST: Tuberculin Skin Test; IGRA: Interferon-Gamma Release Assay; CXR: Chest X-Ray; LTBI: Latent Tuberculosis Infection; TB: Tuberculosis; BCG: Bacillus Calmette-Guérin

#### Costs

Costs for LTBI screening and treatment were derived from the British Columbia Centre for Disease Control (BCCDC) in 2014 (personal communication), and included the costs of tests, drugs, clinician time, and routine monitoring. Adverse event and hospitalization costs during LTBI and TB treatment were determined from the literature [37,40]. The average cost for each TB case, which includes diagnosis, treatment, contact investigation, and adverse events, was estimated from a Canadian report and cost-effectiveness analysis [37,39]. All model costs were inflated to 2016 Canadian dollars ($) using purchasing power parity and are listed in Table 1.

#### Health state utilities

All health state utilities were defined using the SF-6D scores derived from SF-36 responses and were largely informed by a study performed in new migrants to Canada [42–44]. Health state utilities were evaluated for the duration of time in each health state and not subject to fixed duration. The duration of time in the TB health state varied based on whether a patient was or was not under surveillance, as defined by the time from symptom onset to TB diagnosis reported by *Khan et al*. [4] A baseline value of 0.81 was used for all participants without LTBI or TB [42], with adjustments for other health states, where applicable. Table 1 contains all utility values and adjustments.

### Interventions

Several LTBI screening and treatment interventions available in Canada were evaluated, assuming that at each step all migrants evaluated were offered an intervention (i.e. clinician discretion in offering screening and/or treatment was not simulated and no actual data exist on how often LTBI screening is given). LTBI screening interventions included: (1) tuberculin skin test (TST), a test that requires a follow-up visit to be read and uses ≥10mm cut-point for a positive result; (2) interferon-gamma release assay (IGRA), a test that may generate indeterminate results and uses the manufacturer’s recommendation for a positive result and; (3) sequential screening (SEQ), a two-stage approach where those who test positive with a TST are tested with an IGRA—both tests must be positive for the patient to be considered to have LTBI [55,56].

Subsequently, test positive migrants who completed the medical evaluation and initiated treatment were offered one of two LTBI interventions available in Canada: (1) nine-months of isoniazid, which reduces risk of future TB by 93% and; (2) four-months of rifampin, a shorter regimen with higher completion rates, but uncertain efficacy. In general, only those flagged and adhering with post-landing surveillance are offered LTBI interventions upon arrival and Canadian guidelines recommend screening with a TST and subsequent treatment with isoniazid [1]. Thus, LTBI screening with a TST and treatment with isoniazid in the migrant population under surveillance was considered our base case in all cost-effectiveness analyses. A comprehensive table of interventions is located in S3 Table.

### Cost-effectiveness analyses

#### Improving the post-landing surveillance system

In this evaluation, the analysis focused solely on the 2.4% of new migrants normally flagged for post-landing surveillance (N = 6100), as a system is already in place where LTBI interventions can be easily implemented. In the primary analysis, interventions were compared to the base case under real world care conditions. The total number of discounted TB cases (including imported TB cases), costs, and QALYs were calculated for each intervention. The incremental cost-effectiveness ratio (ICER) was calculated for each intervention compared to the base case.

A secondary analysis was performed to determine if improving the cascade of care would be valuable. In this analysis, improving to surveillance adherence to 100%, improving LTBI treatment completion by 30%, and achieving both, was modeled. Based on our WTP threshold ($100,000 per QALY gained), the maximum cost that could be afforded to the healthcare system to implement these improvements was calculated using net monetary benefit (NMB).

#### Implementation of mass post-landing LTBI screening

In this evaluation, the entire 2014 entry cohort is included (N = 260,600). Post-landing LTBI screening was evaluated through step-wise expansion of the post-landing surveillance system based on TB incidence in country of origin (i.e. screen migrants from very high TB incidence countries, screen migrants from countries of high TB incidence or greater, screen migrants from countries of moderate TB incidence or greater, screen all migrants). We modeled this intervention under the assumption that it was implemented as a supplement to the current post-landing surveillance system, therefore, even if migrants were not subject to mass post-landing screening, they could still be flagged for post-landing surveillance. Each intervention was compared to the base case. Adherence with post-landing screening was assumed to be the same as in migrants flagged for surveillance (60.5%). Discounted costs, QALYs, and TB cases (including imported TB cases) were compared to the base case.

### Sensitivity analysis

Uncertainty around model variables was examined using univariate sensitivity analysis and probabilistic sensitivity analysis (PSA). Ranges examined for both univariate and PSA can be found in Table 1. All sensitivity analyses were run for ≥2000 iterations. Results of univariate sensitivity analysis were reported as NMB of the most cost-effective option in our first primary analysis (“Improving the Post-Landing Surveillance System”) compared to the base case. A PSA was performed for each primary analysis to evaluate parameter uncertainty. When model variables came from the literature, relevant distributions were used (e.g. log-normal, beta). Most costs were modeled using relevant triangular distributions due to lack of individual data. In the case of LTBI treatment, extreme costs commonly seen in treatment due to adverse events were accounted for by modeling these separately. In the case of TB treatment, expert opinion was used to develop a relevant gamma distribution. Particular health states were correlated to prevent implausible values during PSA (i.e. patients with active TB will always have a lower utility value than healthy patients). Using the average results of our PSA, efficiency frontiers comparing interventions based on costs and QALYs were developed. Cost-effectiveness acceptability curves were developed based on the probability an intervention provided the most NMB over the ≥2000 iterations run in comparison to the base case. The expected value of perfect information (EVPI) was calculated using the ≥2000 iterations (second-order uncertainty) as our outer sample size and the size of the population evaluated as our inner sample size (first-order uncertainty).

## Results

### Improving the post-landing surveillance system

In the base case scenario, the migrant population under post-landing surveillance (N = 6100) experience, on average, 99.41 cases of TB, incur $3.1 million in costs, and accrue 45,026 QALYs over ten-years (Table 2). Screening with an IGRA and treating with rifampin was dominant in comparison, preventing 4.90 TB cases (a 4.9% reduction), adding 4.0 QALYs, and saving $353,013. While treating with isoniazid was also dominant, preventing more TB cases (6.71) and adding more QALYs (4.8), it only provided an incremental NMB of $676,330 compared to the incremental NMB provided by rifampin treatment of $753,658, making rifampin the preferred treatment.

**Table 2:** Discounted results of base case analysis of the population under medical surveillance.

Intervention	Total TB Cases (Change from Reference)	Population Costs ($) (Change from Reference)	Population QALYs (Change from Reference)	Incremental Cost per TB Case Prevented ($)	Incremental Cost per QALY gained ($)
TST/INH (Reference)	99.41	3,137,675	45,026.1	-	-
TST/RIF	100.58 (1.17)	2,914,913 (-222,762)	45,025.4 (-0.7)	191,236†	312,952†
IGRA/INH	92.70 (-6.71)	2,946,383 (-191,292)	45,030.9 (4.8)	Dominant	Dominant
IGRA/RIF	94.51 (-4.90)	2,784,661 (-353,014)	45,030.1 (4.0)	Dominant	Dominant
SEQ/INH	100.58 (1.17)	2,853,649 (-284,026)	45,025.8 (-0.3)	242,882†	1,064,235†
SEQ/RIF	101.73 (2.32)	2,756,316 (-381,359)	45,024.8 (-1.3)	164,292†	308,919†
No Intervention	113.56 (14.15)	2,616,436 (-521,239)	45,016.0 (-10.1)	36,836†	51,581†

^†^The result falls in Quadrant III, worse outcomes with lower cost. The result should be interpreted inversely

TB: Tuberculosis, QALYs: Quality Adjusted Life Years; TST: Tuberculin skin test; IGRA: interferon-gamma release assay; SEQ: sequential screening; INH: isoniazid; RIF: rifampin

A NMB of $1,098,510 resulted from improving treatment completion by 30% and $1,557,078 resulted when ensuring 100% adherence with surveillance when screening with an IGRA and treating with rifampin. If both of these improvements could be achieved, a NMB of $2,068,246 resulted. While investing in improving post-landing adherence added more QALYs and prevented more TB cases, the added costs of screening and treatment limit the proportional NMB of such an intervention (Table 3).

**Table 3:** Results of LTBI cascade of care improvements in the population under medical surveillance.

Intervention	Change* in TB Cases	Change* in Population Costs ($)	Change* in Population QALYs	Amount Available to Invest per Cohort at WTP of $100,000 per QALY Gained ($)
Improve Treatment Completion by 30%				
TST/INH	-2.13	38,308	1.7	127,358
TST/RIF	-1.32	-242,145	1.2	366,737
IGRA/INH	-9.70	-188,394	7.5	941,790
IGRA/RIF	-8.73	-407,861	6.9	1,098,510
SEQ/INH	-0.38	-277,563	0.5	331,006
SEQ/RIF	0.30	-407,679	0.1	420,370
Perfect Adherence with Surveillance				
TST/INH	-9.40	430,200	7.7	339,075
TST/RIF	-7.84	54,442	6.0	549,260
IGRA/INH	-20.43	108,094	14.6	1,351,074
IGRA/RIF	-17.79	-161,603	14.0	1,557,078
SEQ/INH	-7.11	-35,082	6.3	660,236
SEQ/RIF	-5.58	-197,657	4.6	660,570
Perfect Adherence with Surveillance and Improve Treatment Completion by 30%				
TST/INH	-12.88	494,333	10.5	559,007
TST/RIF	-11.75	28,971	8.7	836,791
IGRA/INH	-25.44	110,840	18.4	1,733,599
IGRA/RIF	-23.90	-246,880	18.2	2,068,246
SEQ/INH	-10.19	-34,954	8.6	893,888
SEQ/RIF	-9.27	-249,568	7.1	956,461

*Change from Reference Intervention: 99.41 Cases of TB, $3,137,675 Population Costs, and 45,026.1 Population QALYs

TB: Tuberculosis, QALYs: Quality Adjusted Life Years; TST: Tuberculin skin test; IGRA: interferon-gamma release assay; SEQ: sequential screening; INH: isoniazid; RIF: rifampin; WTP: willingness-to-pay; LTBI: latent tuberculosis infection

### Implementation of mass post-landing LTBI screening

The most effective intervention to implement for post-landing LTBI screening of every new permanent resident to reduce TB cases was to screen with an IGRA and treat with isoniazid, preventing 125.99 TB cases (a 21.8% reduction) at a cost of $169,986 per TB case prevented; screening with an IGRA and treating with rifampin added the most QALYs, with an additional 78.3 QALYs at a cost of $207,328 per QALY gained. The most cost-effective intervention, was to limit post-landing LTBI screening to every new migrant from countries with a TB incidence ≥30 per 100,000 and screen with an IGRA, followed by treatment with rifampin, which had a cost per TB case prevented of $114,840 and $138,484 per QALY gained (Table 4).

**Table 4:** Results of expanding post-landing LTBI screening based on TB incidence in country of origin.

Intervention	Total TB Cases (Change from Reference)	Population Costs ($) (Change from Reference)	Population QALYs (Change from Reference)	Incremental Cost per TB Case Prevented ($)	Incremental Cost per QALY gained ($)
Reference (TST/INH in those under surveillance)	578.18	13,479,792	1,930,729.6	-	-
Screen all from ≥200 per 100,000					
TST/INH	545.01 (-33.17)	22,413,667 (8,933,875)	1,930,760.6 (31.0)	269,388	288,550
TST/RIF	548.73 (-29.45)	19,439,848 (5,960,056)	1,930,754.0 (24.4)	202,369	244,489
IGRA/INH	520.03 (-58.15)	21,579,890 (8,100,098)	1,930,768.3 (38.7)	139,305	209,222
IGRA/RIF	524.71 (-53.47)	19,079,482 (5,599,690)	1,930,761.6 (32.0)	104,729	175,131
SEQ/INH	550.38 (-27.80)	18,775,849 (5,296,057)	1,930,739.4 (9.8)	190,545	541,408
SEQ/RIF	554.75 (-23.43)	17,301,425 (3,821,633)	1,930,746.1 (16.5)	163,104	231,661
Screen all from ≥100 per 100,000					
TST/INH	517.00 (-67.18)	29,298,355 (15,818,563)	1,930,760.5 (30.9)	258,590	511,673
TST/RIF	524.11 (-54.07)	24,250,547 (10,770,755)	1,930,765.1 (35.5)	199,218	303,254
IGRA/INH	478.42 (-99.76)	26,783,895 (13,304,103)	1,930,792.0 (62.4)	133,369	213,406
IGRA/RIF	486.92 (-91.26)	22,944,405 (9,464,613)	1,930,793.9 (64.3)	103,714	147,350
SEQ/INH	526.43 (-51.75)	22,326,981 (8,847,189)	1,930,757.1 (27.5)	170,962	321,508
SEQ/RIF	531.95 (-46.23)	20,020,938 (6,541,146)	1,930,763.2 (33.6)	141,505	194,940
Screen all from ≥30 per 100,000					
TST/INH	503.00 (-75.18)	36,534,345 (23,054,553)	1,930,767.6 (38.0)	306,672	607,385
TST/RIF	508.41 (-69.77)	29,309,392 (15,829,600)	1,930,784.1 (54.5)	226,898	290,511
IGRA/INH	454.91 (-123.27)	30,992,637 (17,512,845)	1,930,807.5 (77.9)	142,079	224,739
IGRA/RIF	466.44 (-111.74)	26,311,297 (12,831,505)	1,930,822.3 (92.7)	114,840	138,484
SEQ/INH	513.32 (-64.86)	25,263,671 (11,783,879)	1,930,775.8 (46.2)	181,693	255,395
SEQ/RIF	519.41 (-58.77)	22,418,827 (8,939,035)	1,930,774.6 (45.0)	152,121	198,819
Screen all new migrants					
TST/INH	501.14 (-77.04)	42,460,450 (29,980,658)	1,930,780.8 (51.2)	376,180	566,155
TST/RIF	506.55 (-71.63)	33,689,173 (20,209,381)	1,930,778.2 (48.6)	282,148	415,606
IGRA/INH	452.19 (-125.99)	34,895,981 (21,416,189)	1,930,803.5 (73.9)	169,986	289,838
IGRA/RIF	463.67 (-114.51)	29,720,266 (16,240,474)	1,930,808.0 (78.4)	141,825	207,328
SEQ/INH	510.88 (-67.30)	27,535,513 (14,055,721)	1,930,778.0 (48.4)	208,859	290,448
SEQ/RIF	518.21 (-59.97)	24,497,307 (11,017,515)	1,930,770.3 (40.7)	183,724	270,562

TB: Tuberculosis, QALYs: Quality Adjusted Life Years; TST: Tuberculin skin test; IGRA: interferon-gamma release assay; SEQ: sequential screening; INH: isoniazid; RIF: rifampin;

### Sensitivity analysis

In univariate sensitivity analysis the base case intervention was compared to IGRA followed by rifampin, in migrants under surveillance. Extending the time horizon had the most significant impact in favor of IGRA followed by rifampin, as the incremental NMB increased by over $1.2 million if extended to 50 years. Reducing the effectiveness of a full course of rifampin to 50% had the most significant impact against IGRA followed by rifampin, reducing the incremental NMB by over $600,000. The decision to favor IGRA screening followed by rifampin treatment over the base case, however, was very robust as no single parameter change resulted in the base case having a higher NMB. Further univariate sensitivity analysis results can be found in S2 Text, S4 Table and S1 Fig.

**Fig 3:**
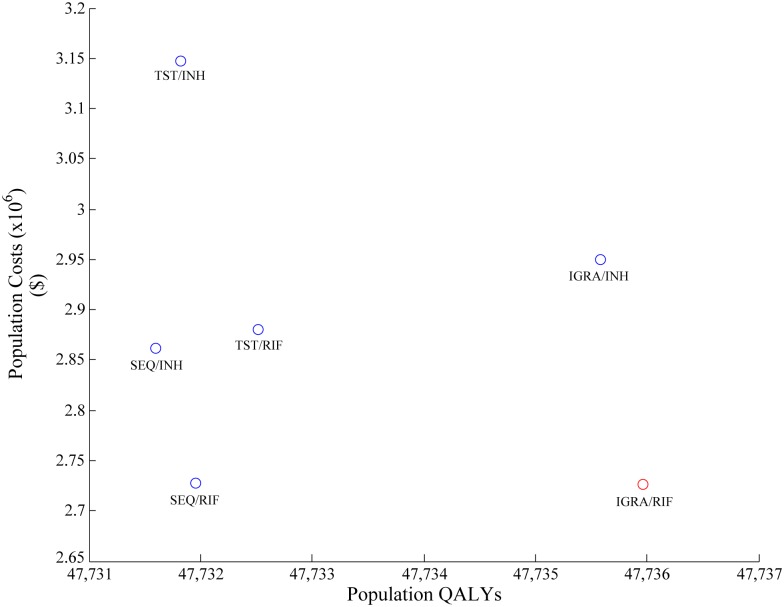
Efficiency frontier of population QALYs vs. population costs in the 2014 population of migrants under post-landing surveillance. The frontier is read from *left to right*, with interventions connected if they fall on the frontier. Interventions subsequent to the initial intervention have an increased cost, but an increased benefit, and represent the next best value at increasing funding thresholds. The slope between two connected interventions represents cost-effectiveness: a steeper slope represents poorer cost-effectiveness between interventions, while a shallow slope represents better cost-effectiveness. QALY: quality adjusted life year; SEQ: sequential screening; TST: tuberculin skin test; IGRA: interferon-gamma release assay; INH: isoniazid; RIF: rifampin.

**Fig 4:**
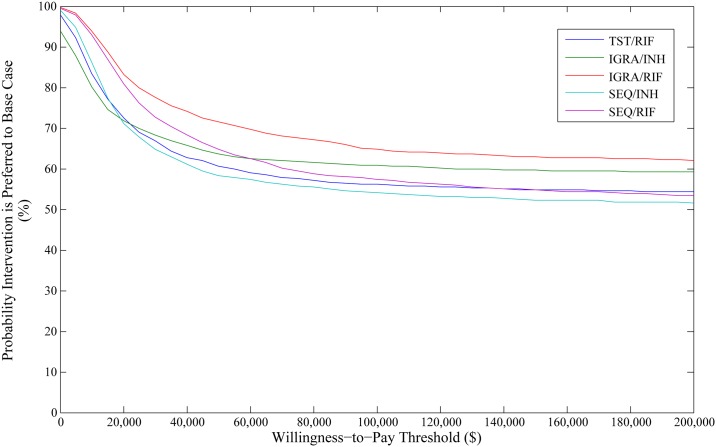
Cost-effectiveness acceptability curve for cost per QALY gained in the 2014 population of migrants under post-landing surveillance. The graph demonstrates the probability an intervention is cost-effective at various willingness-to-pay thresholds in relation to the base case intervention. QALY: quality adjusted life year; SEQ: sequential screening; TST: tuberculin skin test; IGRA: interferon-gamma release assay; INH: isoniazid; RIF: rifampin.

In PSA of our primary analysis of migrants under surveillance, screening with an IGRA followed by rifampin treatment was the dominant option, resulting in the lowest cost, minimizing TB cases and maximizing QALYs. Screening sequentially or with a TST did not fall on the frontier (Fig 3). Due to the base case being the most expensive option, probabilities of interventions being cost-effective fell as WTP thresholds increased. Use of IGRA followed by rifampin had a probability of being cost-effective of 64.9% at a WTP of $100,000 per QALY gained, however increasing the WTP impacted the probability minimally (Fig 4). It was determined that the choice of IGRA followed by rifampin over the base case resulted in an EVPI of $610,102.

**Fig 5:**
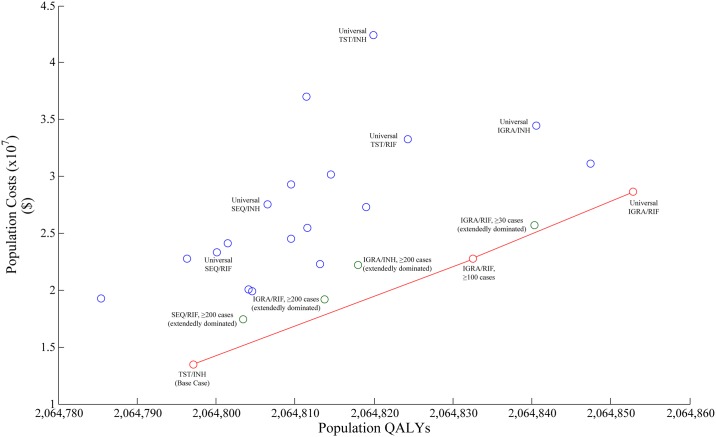
Efficiency frontier of population QALYs vs. population costs in the complete 2014 cohort of migrants. The frontier is read from *left to right*, with interventions connected if they fall on the frontier. Interventions subsequent to the initial intervention have an increased cost, but an increased benefit, and represent the next best value at increasing funding thresholds. The slope between two connected interventions represents cost-effectiveness: a steeper slope represents poorer cost-effectiveness between interventions, while a shallow slope represents better cost-effectiveness. QALY: quality adjusted life year; SEQ: sequential screening; TST: tuberculin skin test; IGRA: interferon-gamma release assay; INH: isoniazid; RIF: rifampin.

**Fig 6:**
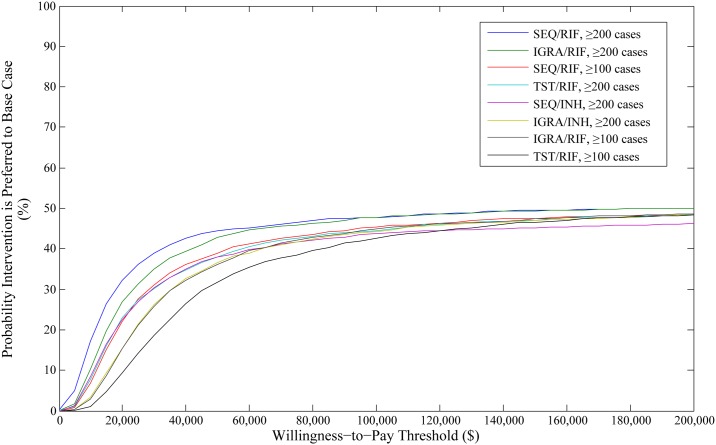
Cost-effectiveness acceptability curve for cost per QALY gained in the complete 2014 cohort of migrants. The graph demonstrates the probability an intervention is cost-effective at various willingness-to-pay thresholds in relation to the base case intervention. QALY: quality adjusted life year; SEQ: sequential screening; TST: tuberculin skin test; IGRA: interferon-gamma release assay; INH: isoniazid; RIF: rifampin.

In PSA of our analysis in the total migrant cohort, it was found that the base case provided the best value for the least investment. In efficiency frontier analysis, screening with an IGRA followed by treatment with rifampin in all migrants maximized QALYs. No TST screening intervention fell on the frontier (Fig 5). Use of IGRA followed by rifampin in migrants from countries ≥30 cases per 100,000, the most cost-effective option in deterministic analysis, had a probability of being cost-effective of 43.3% at a WTP of $100,000 per QALY, however use of sequential screening followed by rifampin in migrants from countries ≥200 cases per 100,000 had the highest probability of being cost-effective at this threshold of 47.8% (Fig 6). In EVPI analysis, it was found that the decision to remain using our base case intervention compared to use of an IGRA followed by rifampin in migrants from countries ≥30 cases per 100,000, resulted in an EVPI of $12,873,338.

Further PSA results focusing on TB cases can be found in S3 Text, S5 and S6 Tables, S2 and S3 Figs.

## Discussion

The current post-landing TB surveillance system is not effective in achieving the desired declines in TB incidence in Canada. To improve LTBI diagnosis and treatment in new migrants flagged for post-landing surveillance, screening with an IGRA followed by rifampin treatment provides an overall lower cost to the healthcare system, with a reduction in TB cases and an increase in QALYs over a ten-year time horizon. Expanding post-landing LTBI screening and treatment to include all migrants was not cost-effective using any intervention, however had the ability to significantly increase population QALYs and reduce TB cases. Further targeting post-landing LTBI interventions by TB incidence in country of origin significantly improved cost-effectiveness, yet ICERs still remained above WTP thresholds.

In Canada, the current post-landing TB surveillance system was developed to focus on identifying those at highest risk of TB immediately after arrival and was never intended to be a platform where LTBI identification was a priority. Our analysis shows that using this system to screen for LTBI would not significantly impact longitudinal TB cases, even when improving adherence with surveillance and LTBI treatment. In the present system, gaps in the LTBI cascade of care result in <15% of migrants who may benefit from treatment actually completing LTBI therapy [9]. Ensuring 100% adherence to post-landing surveillance and improving completion of therapy by 30%, still less than one-third of migrants would complete LTBI therapy. Our data and others suggest that there is significant room for investment in improving treatment adherence [57], yet it is evident that filling gaps at each step of the cascade of care is crucial to achieving significant reductions in TB incidence.

Our analysis shows that LTBI screening decisions guided solely by TB incidence in country of origin are not specific enough to be cost-effective—it is clear that further targeted screening will be necessary. It is likely that determining the socioeconomic factors that underlie TB infection in migrant populations will be necessary to cost-effectively target LTBI screening and treatment. A previous analysis [12] examined LTBI screening in people with co-morbidities such as silicosis, renal disease, and diabetes and found this targeting not to be cost-effective. Evaluation in migrant populations where LTBI prevalence is significantly higher may lead to a different conclusion, however.

Previous economic analyses have been performed to assess the cost-effectiveness of mass post-landing screening of new migrants to low-incidence countries, several of which have been highlighted in systematic reviews [58–60]. In an analysis by Dasgupta et al. [11], post-landing surveillance was evaluated for its ability to prevent TB cases in new immigrants to Montreal. The analysis found that in this setting, post-landing surveillance prevented 1.9 cases of TB per 1000 new immigrants identified, for an incremental cost of $65,126 per TB disease prevented, slightly different from our results of 2.3 cases prevented per 1000 immigrants identified for post-landing surveillance ($36,837 per TB disease prevented).

Oxlade and colleagues evaluated IGRA and TST screening in new immigrants from varying TB incidence groups [56] and found that CXR at entry was cost-effective in immigrants from intermediate-to-high TB incidence countries, while IGRAs and TSTs were not cost-effective. This is in agreement with our findings, where no screening method was cost-effective in a mass LTBI screening scenario, regardless of TB incidence in country of origin.

An evaluation performed by Khan et al [10] examined mass LTBI interventions in the United States and found it to be net saving. This evaluation, however, assumed no dropout during screening and/or treatment, which would be incredibly difficult to implement in practice. Finally, Linas et al [12], examined LTBI screening in new migrants and found it to be cost-effective, assuming low rates of dropout in modeled portions of the LTBI cascade of care and different diagnostic test performance in their model.

Our analysis is the first to comprehensively model gaps in the LTBI cascade of care. We have shown that these gaps limit the effectiveness of any mass intervention to target LTBI, with <20% of those who can potentially benefit from LTBI therapy completing a course. This model is also the first to estimate the prevalence of LTBI and migrant TB in new migrant cohorts to Canada based on incident TB, rather than TST reactivity. Further, the use of DES allowed for varying times in different health states for different migrants; this allows for more accurate simulation of real world utility data and the impact each health state has on total quality of life. Our accurate representation of “healthy” utility for new migrants limits our bias of LTBI interventions away from the null, as is seen when healthy utility is assumed to be one [61]. A significant strength of this model was that it was specifically calibrated to Canadian immigration data and the TB profile of new immigrants to Ontario, which has the ability to effectively inform policy decisions in Canada. Further, these results may be generalizable to other low TB incidence countries that also use CXR to identify new migrants at high-risk for TB and have a similar migrant profile to Canada.

Our study has several limitations. The proportion of remote infections was tied to intervention; in essence, if fewer TB cases occurred due to reactivation, so too did TB cases due to remote transmission, which may not reflect reality where reductions in TB reactivation likely don’t exactly match reductions in TB transmission. This model assumes that all migrants reporting to the clinic are offered LTBI screening; which is unlikely. Furthermore, for migrants referred due to a previous diagnosis of TB, LTBI diagnostic tests may not be reliable due to a lasting immune response, however it was not possible to determine from our database how many migrants fell into this category; thus it was unaccounted for in our model although we do not think many individuals would fall into this category and making a real difference in the model results. Moreover, we assumed that dropout at each step of the care cascade is random, which may not reflect reality, as some patients will never be offered therapy due to age, co-morbid conditions, or feasibility. Nevertheless, this was a necessary assumption in our model due to the level of evidence available. Finally, this model did not consider co-morbid conditions that may increase risk of TB, however the studies informing our rate of reactivation were derived from diverse populations and should approximate a population-wide reactivation rate.

Future economic analyses of LTBI interventions in migrant populations should focus on varying the timing of screening and/or how to target screening. Research into LTBI screening during pre-immigration medical exams could potentially be highly valuable as a tool for post-landing follow-up. Furthermore, targeting screening post-landing based on a combination of co-morbidities and demographic variables can potentially make strong predictions about future TB risk in individuals.

## Conclusion

Screening new migrants flagged for post-landing surveillance with an IGRA followed by treatment with rifampin was dominant compared to the base case of TST followed by isoniazid. Expanding LTBI screening to all new migrants was cost-prohibitive. Future research should investigate the cost-effectiveness of LTBI screening based on socioeconomic factors and co-morbid conditions.

## Supporting information

S1 Text Model optimization. (DOCX)

S2 Text Results of univariate sensitivity analysis. (DOCX)

S3 Text Results of probabilistic sensitivity analysis. (DOCX)

S1 Table Model optimization targets. (DOCX)

S2 Table Final results of optimization. (DOCX)

S3 Table Interventions evaluated. (DOCX)

S4 Table Base estimates and univariate analysis range. (DOCX)

S5 Table Probability (%) an intervention was cost-effective compared to the base case in the total migrant population. (DOCX)

S6 Table Probability (%) an intervention was cost-effective compared to the base case in the population under medical surveillance. (DOCX)

S1 Formula Chance of remote transmission occurring per reactivation. (DOCX)

S1 Fig Results of the univariate sensitivity analysis using QALYs as effectiveness measure. The figure is ordered in the direction of absolute effect (i.e. not in order of direction of effect) on the net monetary benefit in relation to what was calculated in deterministic analysis. QALY: quality adjusted life years; TST: tuberculin skin test; IGRA: interferon-gamma release assay; LTBI: latent tuberculosis infection; TB: tuberculosis; CXR: chest x-ray.
(TIF)

S2 Fig Efficiency frontier of population TB cases vs. population costs in the 2014 population of migrants under post-landing surveillance. The frontier is read from *left to right*, with interventions connected if they fall on the frontier; the x-axis is in the reverse direction for ease of understanding. Interventions subsequent to the initial intervention have an increased cost, but an increased benefit (reduction in TB cases), and represent the next best value at increasing funding thresholds. The slope between two connected interventions represents cost-effectiveness: a steeper slope represents poorer cost-effectiveness between interventions, while a shallow slope represents better cost-effectiveness. Intervention(s) that are extendedly dominated have a higher cost-per TB case prevented and more population TB cases than the subsequent intervention on the frontier and are therefore less efficient. SEQ: Sequential Screening; RIF: rifampin therapy; INH: isoniazid therapy; TST: tuberculin skin test; IGRA: interferon-gamma release assay; TB: tuberculosis.
(TIF)

S3 Fig Efficiency frontier of population TB cases vs. population costs in the complete 2014 cohort of migrants. The frontier is read from *left to right*, with interventions connected if they fall on the frontier; the x-axis is in the reverse direction for ease of understanding. Interventions subsequent to the initial intervention have an increased cost, but an increased benefit (reduction in TB cases), and represent the next best value at increasing funding thresholds. The slope between two connected interventions represents cost-effectiveness: a steeper slope represents poorer cost-effectiveness between interventions, while a shallow slope represents better cost-effectiveness. Intervention(s) that are extendedly dominated have a higher cost-per TB case prevented and more population TB cases than the subsequent intervention on the frontier and are therefore less efficient. SEQ: Sequential Screening; RIF: rifampin therapy; INH: isoniazid therapy; TST: tuberculin skin test; IGRA: interferon-gamma release assay; TB: tuberculosis.
(TIF)

S1 Checklist Consolidated health economic evaluation reporting standards checklist. (PDF)

## References

[1] Canadian Tuberculosis Standards, 7th Edition Public Health Agency of Canada 2013 http://www.phac-aspc.gc.ca/tbpc-latb/pubs/tb-canada-7/index-eng.php [accessed May 11, 2017].

[2] Roth D, Johnston J, Cook V. TB in foreign-born patients. BC Med J. 2012;54(8): 387–8.

[3] Long R, Sutherland K, Kunimoto D, Cowie R, Manfreda J. The epidemiology of tuberculosis among foreign-born persons in Alberta, Canada, 1989–1998: identification of high risk groups. Int J Tuberc Lung Dis. 2002;6: 615–21. 12102301

[4] Khan K, Hirji MM, Miniota J, Hu W, Wang J, Gardam M, et al Domestic impact of tuberculosis screening among new immigrants to Ontario, Canada. Can Med Assoc J. 2015;187(16): E473–81. 26416993 10.1503/cmaj.150011 PMC4627893

[5] Chin DP, DeRiemer K, Small PM, de Leon AP, Steinhart R, Schecter GF, et al Differences in contributing factors to tuberculosis incidence in U.S. -born and foreign-born persons. Am J Respir Crit Care Med. 1998;158: 1797–803. doi: 10.1164/ajrccm.158.6.9804029 9847270 10.1164/ajrccm.158.6.9804029

[6] Alexander DC, Guthrie JL, Pyskir D, Maki A, Kurepina N, Kreiswirth BN, et al Mycobacterium tuberculosis in Ontario, Canada: Insights from IS6110 restriction fragment length polymorphism and mycobacterial interspersed repetitive-unit-variable-number tandem-repeat genotyping. J Clin Microbiol. 2009;47: 2651–4. doi: 10.1128/JCM.01946-08 19494075 10.1128/JCM.01946-08 PMC2725669

[7] Shea KM, Kammerer JS, Winston CA, Navin TR, Horsburgh CR. Estimated rate of reactivation of latent tuberculosis infection in the United States, overall and by population subgroup. Am J Epidemiol. 2014;179(2):216–25. doi: 10.1093/aje/kwt246 24142915 10.1093/aje/kwt246 PMC5547435

[8] International Union Against Tuberculosis Committee on Prophylaxis. Efficacy of various durations of isoniazid preventive therapy for tuberculosis: five years of follow-up in the IUAT trial. Bull World Health Organ. 1982;60: 555–64. 6754120 PMC2536088

[9] Alsdurf H, Hill PC, Matteelli A, Getahun H, Menzies D. The cascade of care in diagnosis and treatment of latent tuberculosis infection: a systematic review and meta-analysis. Lancet Infect Dis. 2016;16(11): 1269–78. doi: 10.1016/S1473-3099(16)30216-X 27522233 10.1016/S1473-3099(16)30216-X

[10] Khan K, Muennig P, Behta M, Zivin JG. Global Drug-Resistance Patterns and the Management of Latent Tuberculosis Infection in Immigrants to the United States. N Engl J Med. 2002;347: 1850–9. doi: 10.1056/NEJMsa021099 12466510 10.1056/NEJMsa021099

[11] Dasgupta K, Schwartzman K, Marchand R, Tennenbaum TN, Brassard P, Menzies D. Comparison of Cost-Effectiveness of Tuberculosis Screening of Close Contacts and Foreign-Born Populations. Am J Respir Crit Care Med. 2000;162: 2079–86. doi: 10.1164/ajrccm.162.6.2001111 11112118 10.1164/ajrccm.162.6.2001111

[12] Linas BP, Wong AY, Freedberg KA, Horsburgh CR. Priorities for screening and treatment of latent tuberculosis infection in the United States. Am J Respir Crit Care Med. 2011;184: 590–601. doi: 10.1164/rccm.201101-0181OC 21562129 10.1164/rccm.201101-0181OC PMC3175546

[13] Canada Facts and Figures: Immigrant Overview Permanent Residents 2014. Government of Canada: Immigration, Refugees and Citizenship Canada. 2015. http://publications.gc.ca/site/eng/9.512569/publication.html [accessed May 11, 2017]

[14] BCG World Atlas, 2nd Edition. 2017. http://www.bcgatlas.org [accessed May 11, 2017]

[15] WHO-UNICEF Estimates of BCG Coverage. World Health Organization. 2017. http://apps.who.int/immunization_monitoring/globalsummary/timeseries/tswucoveragebcg.html [accessed May 11, 2017]

[16] Walter ND, Painter J, Parker M, Lowenthal P, Flood J, Fu Y, et al Persistent latent tuberculosis reactivation risk in United States immigrants. Am J Respir Crit Care Med. 2014;189: 88–95. 24308495 10.1164/rccm.201308-1480OC PMC3919127

[17] Aldridge RW, Zenner D, White PJ, Williamson EJ, Muzyamba MC, Dhavan P, et al Tuberculosis in migrants moving from high-incidence to low-incidence countries: a population-based cohort study of 519 955 migrants screened before entry to England, Wales, and Northern Ireland. Lancet. 2016;388: 2510–8. doi: 10.1016/S0140-6736(16)31008-X 27742165 10.1016/S0140-6736(16)31008-X PMC5121129

[18] Ferebee SH. Controlled chemoprophylaxis trials in tuberculosis. A general review. Bibl Tuberc. 1970;26: 28–106. 4903501

[19] Comstock GW, Livesay VT, Woolpert SF. The prognosis of a positive tuberculin reaction in childhood and adolescence. Am J Epidemiol. 1974;99(2): 131–8. 4810628 10.1093/oxfordjournals.aje.a121593

[20] Ferebee SH, Mount FW, Murray FJ, Livesay VT. A controlled trial of isoniazid prophylaxis in mental institutions. Am Rev Respir Dis. 1963;88: 161–75. 14045220 10.1164/arrd.1963.88.2.161

[21] Edwards LB, Livesay VT, Acquaviva FA, Palmer CE. Height, weight, tuberculosis infection, and tuberculosis disease. Arch Environ Health. 1971;22(1): 106–12. 4992917 10.1080/00039896.1971.10665820

[22] Chan IHY, Kaushik N, Dobler CC. Post-migration follow-up of migrants identified to be at increased risk of developing tuberculosis at pre-migration screening: a systematic review and meta-analysis. Lancet Infect Dis. 2017;0 doi: 10.1016/S1473-3099(17)30194-9 10.1016/S1473-3099(17)30194-9 28410979

[23] Guidelines for the Economic Evaluation of Health Technologies: Canada (4th Edition, 2017). Canadian Agency for Drugs and Technologies in Health. 2017. https://www.cadth.ca/guidelines-economic-evaluation-health-technologies-canada-4th-edition [accessed August 16, 2017].

[24] Laupacis A, Feeny D, Detsky AS, Tugwell PX. How attractive does a new technology have to be to warrant adoption and utilization? Tentative guidelines for using clinical and economic evaluations. Can Med Assoc J. 1992;146: 473–81. 1306034 PMC1488412

[25] Marseille E, Larson B, Kazi DS, Kahn JG, Rosen S. Thresholds for the cost-effectiveness of interventions: alternative approaches. Bull World Health Organ. 2015;93:118–24. doi: 10.2471/BLT.14.138206 25883405 10.2471/BLT.14.138206 PMC4339959

[26] Neumann PJ, Cohen JT, Weinstein MC. Updating cost-effectiveness—the curious resilience of the $50,000-per-QALY threshold. N Engl J Med. 2014;371(9):796–7. doi: 10.1056/NEJMp1405158 25162885 10.1056/NEJMp1405158

[27] Sanders GD, Neumann PJ, Basu A, Brock DW, Feeny D, Krahn M, et al Recommendations for Conduct, Methodological Practices, and Reporting of Cost-effectiveness Analyses: Second Panel on Cost-Effectiveness in Health and Medicine. JAMA. 2016;316(10):1093–1103. doi: 10.1001/jama.2016.12195 27623463 10.1001/jama.2016.12195

[28] Life Tables, Canada, Provinces and Territories 2010–2012. Government of Canada: Statistics Canada. 2016. http://www5.statcan.gc.ca/olc-cel/olc.action?objId=84-537-X&objType=2&lang=en&limit=0 [accessed May 11, 2017].

[29] Campbell JR, Krot J, Elwood K, Cook V, Marra F. A systematic review on TST and IGRA tests used for diagnosis of LTBI in immigrants. Mol Diagn Ther. 2015;19: 9–24. doi: 10.1007/s40291-014-0125-0 25579159 10.1007/s40291-014-0125-0

[30] Menzies D, Pai M, Comstock G. Meta-analysis: new tests for the diagnosis of latent tuberculosis infection: areas of uncertainty and recommendations for research. Ann Intern Med. 2007;146: 340–54. 17339619 10.7326/0003-4819-146-5-200703060-00006

[31] Pai M, Zwerling A, Menzies D. Systematic review: T-cell-based assays for the diagnosis of latent tuberculosis infection: an update. Ann Intern Med. 2008;149: 177–84. 18593687 10.7326/0003-4819-149-3-200808050-00241 PMC2951987

[32] Palmer CE, Jablon S, Edwards PQ. Tuberculosis morbidity of young men in relation to tuberculin sensitivity and body build. Am Rev Tuberc. 1957;76(4): 517–39. 13470303 10.1164/artpd.1957.76.4.517

[33] Comstock GW, Palmer CE. Long-term results of BCG vaccination in the southern United States. Am Rev Respir Dis. 1966;93(2): 171–83. 5908081 10.1164/arrd.1966.93.2.171

[34] Horsburgh CR Jr. Priorities for the treatment of latent tuberculosis infection in the United States. N Engl J Med. 2004;350: 2060–67. doi: 10.1056/NEJMsa031667 15141044 10.1056/NEJMsa031667

[35] Horsburgh CR Jr., O’Donnell M, Chamblee S, Moreland JL, Johnson J, Marsh BJ, et al Revisiting Rates of Reactivation Tuberculosis: A Population-based Approach. Am J Respir Crit Care Med. 2010;182(3): 420–5. doi: 10.1164/rccm.200909-1355OC 20395560 10.1164/rccm.200909-1355OC PMC2921602

[36] Campbell JR, Chen W, Johnston J, Cook V, Elwood K, Krot J, Marra F. Latent tuberculosis infection screening in immigrants to low-incidence countries: a meta-analysis. Mol Diagn Ther. 2015;19(2): 107–17. doi: 10.1007/s40291-015-0135-6 25851739 10.1007/s40291-015-0135-6

[37] Marra F, Marra CA, Sadatsafavi M, Morán-Mendoza O, Cook V, Elwood RK, et al Cost-effectiveness of a new interferon-based blood assay, QuantiFERON-TB Gold, in screening tuberculosis contacts. Int J Tuberc Lung Dis. 2008;12: 1414–24. 19017451

[38] Tan MC, Marra CA, Sadatsafavi M, Marra F, Morán-Mendoza O, Moadebi S, et al Cost-effectiveness of LTBI treatment for TB contacts in British Columbia. Value Health. 2008;11: 842–52. doi: 10.1111/j.1524-4733.2008.00334.x 18489519 10.1111/j.1524-4733.2008.00334.x

[39] Menzies D, Lewis M, Oxlade O. Costs for tuberculosis care in Canada. Can J Public Health. 2008;99: 391–6. 19009923 10.1007/BF03405248 PMC6976239

[40] Holland DP, Sanders GD, Hamilton CD, Stout JE. Costs and cost-effectiveness of four treatment regimens for latent tuberculosis infection. Am J Respir Crit Care Med. 2009;179: 1055–60. doi: 10.1164/rccm.200901-0153OC 19299495 10.1164/rccm.200901-0153OC PMC2689913

[41] Fassbender K, Fainsinger RL, Carson M, Finegan BA. Cost trajectories at the end of life: the Canadian experience. J Pain Symptom Manage. 2009;38: 75–80. doi: 10.1016/j.jpainsymman.2009.04.007 19615630 10.1016/j.jpainsymman.2009.04.007

[42] Bauer M, Ahmed S, Benedetti A, Greenaway C, Lalli M, Leavens A, et al The impact of tuberculosis on health utility: a longitudinal cohort study. Qual Life Res. 2015;24: 1337–49. doi: 10.1007/s11136-014-0858-6 25391490 10.1007/s11136-014-0858-6

[43] Guo N, Marra CA, Marra F, Moadebi S, Elwood RK, Fitzgerald JM. Health state utilities in latent and active tuberculosis. Value Health. 2008;11: 1154–61. doi: 10.1111/j.1524-4733.2008.00355.x 18489493 10.1111/j.1524-4733.2008.00355.x

[44] Marra CA, Marra F, Colley L, Moadebi S, Elwood RK, Fitzgerald JM. Health-related quality of life trajectories among adults with tuberculosis: differences between latent and active infection. Chest. 2008;133: 396–403. doi: 10.1378/chest.07-1494 18198260 10.1378/chest.07-1494

[45] Bettache N, Sant N, Schwartzman K, Cnossen S, Sandoe A, Assayag D, et al Effectiveness Of Pre-Immigration Screening And Post-Arrival Surveillance To Detect Active And Latent Tuberculosis In The Foreign Born: A Systematic Review And Meta-Analysis. Am J Respir Crit Care Med. 2012;185: A6507.

[46] Aspler A, Long R, Trajman A, Dion M-J, Khan K, Schwartzman K, et al Impact of treatment completion, intolerance and adverse events on health system costs in a randomised trial of 4 months rifampin or 9 months isoniazid for latent TB. Thorax. 2010;65: 582–7. doi: 10.1136/thx.2009.125054 20627913 10.1136/thx.2009.125054

[47] Pina JM, Clotet L, Ferrer A, Sala MR, Garrido P, Salleras L, et al Cost-effectiveness of rifampin for 4 months and isoniazid for 9 months in the treatment of tuberculosis infection. Eur J Clin Microbiol Infect Dis. 2013;32: 647–55. doi: 10.1007/s10096-012-1788-2 23238684 10.1007/s10096-012-1788-2

[48] Page KR, Sifakis F, Montes de Oca R, Cronin WA, Doherty MC, Federline L, et al Improved adherence and less toxicity with rifampin vs isoniazid for treatment of latent tuberculosis: a retrospective study. Arch Intern Med. 2006;166: 1863–70. doi: 10.1001/archinte.166.17.1863 17000943 10.1001/archinte.166.17.1863

[49] Lardizabal A, Passannante M, Kojakali F, Hayden C, Reichman LB. Enhancement of treatment completion for latent tuberculosis infection with 4 months of rifampin. Chest. 2006;130: 1712–7. doi: 10.1378/chest.130.6.1712 17166986 10.1378/chest.130.6.1712

[50] Menzies D, Dion M-J, Rabinovitch B, Mannix S, Brassard P, Schwartzman K. Treatment completion and costs of a randomized trial of rifampin for 4 months versus isoniazid for 9 months. Am J Respir Crit Care Med. 2004;170: 445–9. doi: 10.1164/rccm.200404-478OC 15172892 10.1164/rccm.200404-478OC

[51] Salpeter SR. Fatal isoniazid-induced hepatitis. Its risk during chemoprophylaxis. West J Med. 1993;159: 560–4. 8279152 PMC1022345

[52] Menzies D, Al Jahdali H, Al Otaibi B. Recent developments in treatment of latent tuberculosis infection. Indian J Med Res. 2011;133: 257–66. 21441678 PMC3103149

[53] Reichman LB, Lardizabal A, Hayden CH. Considering the Role of Four Months of Rifampin in the Treatment of Latent Tuberculosis Infection. Am J Respir Crit Care Med. 2004;170: 832–5. doi: 10.1164/rccm.200405-584PP 15297274 10.1164/rccm.200405-584PP

[54] Jasmer RM, Bozeman L, Schwartzman K, Cave MD, Saukkonen JJ, Metchock B, et al Recurrent tuberculosis in the United States and Canada: relapse or reinfection? Am J Respir Crit Care Med. 2004;170: 1360–6. doi: 10.1164/rccm.200408-1081OC 15477492 10.1164/rccm.200408-1081OC

[55] Roth DZ, Ronald LA, Ling D, Chiang LY, Cook VJ, Morshed MG, et al Impact of interferon-γ release assay on the latent tuberculosis cascade of care: a population-based study. Eur Respir J. 2017;49(3): 1601546 doi: 10.1183/13993003.01546-2016 28331032 10.1183/13993003.01546-2016

[56] Oxlade O, Schwartzman K, Menzies D. Interferon-gamma release assays and TB screening in high-income countries: a cost-effectiveness analysis. Int J Tuberc Lung Dis. 2007;11: 16–26. 17217125

[57] Patel AR, Campbell JR, Sadatsafavi M, Marra F, Johnston JC, Smillie K, Lester RT. Burden of non-adherence to latent tuberculosis infection drug therapy and the potential cost-effectiveness of adherence interventions in Canada: a simulation study. BMJ Open. 2017;7: e015108 doi: 10.1136/bmjopen-2016-015108 28918407 10.1136/bmjopen-2016-015108 PMC5640098

[58] Campbell JR, Sasitharan T, Marra F. A Systematic Review of Studies Evaluating the Cost Utility of Screening High-Risk Populations for Latent Tuberculosis Infection. Appl Health Econ Health Policy. 2015;13: 325–40. doi: 10.1007/s40258-015-0183-4 26129810 10.1007/s40258-015-0183-4

[59] Auguste P, Tsertsvadze A, Court R, Pink J. A systematic review of economic models used to assess the cost-effectiveness of strategies for identifying latent tuberculosis in high-risk groups. Tuberculosis. 2016;99: 81–91. doi: 10.1016/j.tube.2016.04.007 27450009 10.1016/j.tube.2016.04.007

[60] Zammarchi L, Casadei G, Strohmeyer M, Bartalesi F, Liendo C, Matteelli A, et al A scoping review of cost-effectiveness of screening and treatment for latent tuberculosis infection in migrants from high-incidence countries. BMC Health Serv Res. 2015;15: 412 doi: 10.1186/s12913-015-1045-3 26399233 10.1186/s12913-015-1045-3 PMC4581517

[61] Ara R, Brazier JE. Populating an economic model with health state utility values: moving toward better practice. Value Health. 2010;13(5):509–18. doi: 10.1111/j.1524-4733.2010.00700.x 20230546 10.1111/j.1524-4733.2010.00700.x

