# Supplementary material for: Cost-effectiveness of post-landing latent tuberculosis infection control strategies in new migrants to Canada
Source: PLoS One. 2017 Oct 30;12(10):e0186778. doi: 10.1371/journal.pone.0186778 (PMC5662173; doi:10.1371/journal.pone.0186778)
Supplement: S1 Text — (DOCX) [file pone.0186778.s001.docx]

**Model Optimization**

To begin model optimization, latent tuberculosis infection (LTBI) prevalence was estimated based on two-year incidence data from Ontario, Canada [1]. Subgroups were defined by tuberculosis (TB) incidence in country of origin (<30 cases per 100,000 population, 30-99 cases, 100-199 cases, ≥200 cases). This original data was then extrapolated to the migrant distribution in the 2014 permanent resident cohort [2], which yielded the population and TB statistics presented in **S1 Table**.

LTBI prevalence in those not referred for surveillance was calculated on genotypic data suggesting that 85% of all cases of TB in migrants are reactivation; the remaining cases were due to infection in Canada [3,4]. LTBI reactivation rate was varied within reasonable values [5-9] to align with the prevalence of positive IGRAs as reported in a meta-analysis [10]; a value of 1.1 cases per 1000 person years was selected. LTBI prevalence was calculated as follows:

$${Prev}_{LTBI}=\frac{{TB}_{NR}}{P_{NR}*FU*{LTBI}_{RR}}$$

where Prev_LTBI_ is the prevalence of LTBI, TB_NR_ is the number of TB cases in those not-referred for surveillance, P_NR_ is the total population not referred for surveillance, FU is the follow-up time in years, and LTBI_RR_ is the reactivation rate in those with LTBI per person year.

To estimate the proportion TB cases that were imported (i.e. cases of TB that were likely due to recent infection prior to migration but post medical exam or relapse of previously treated TB) and prevalence of LTBI in those referred for surveillance, data from a cohort of migrants from the Philippines with abnormal chest x-rays was used as a benchmark [11]. In this study, 86% of TB cases in year one were due to imported TB. Due to this being a very high incidence population, data was optimized over a plausible range of 55-85% and had to follow the following assumptions: the prevalence of LTBI in migrants referred for surveillance had to be higher than the prevalence of LTBI in migrants not referred for surveillance and the proportion of TB cases occurring due to imported TB were identical across TB incidence subcategories. During optimization, increased risk of reactivation due to an abnormal chest x-ray (CXR) was assumed to be 3.9 times higher than those with a normal CXR, based on data reported by Aldridge et al [12]. Upon optimization, it was found that an imported TB proportion 70% fulfilled did not violate any assumptions. The results of the optimization are presented in **S2 Table**.

After optimization, an age mix of new migrants was developed based on the reported age distribution by Statistics Canada in 2014 [2] and reported TB age distribution by the British Columbia Centre for Disease Control annual report [13]. It was assumed that all TB incidence categories followed the same age distribution. Age probabilities were adjusted based on various factors, including whether a migrants was flagged for surveillance, had LTBI, or had TB. In the case of a migrant having LTBI, the age mix was adjusted based on reported estimates of LTBI by age [2,10]. In addition, in the case of imported TB, the age mix was adjusted to the age profile of TB cases in British Columbia [8]. For the remaining individuals, the base age mix was adjusted to ensure accuracy to the age profile of the 2014 permanent resident cohort (average age: 29 years old). Bacillus Calmette-Guérin (BCG) vaccination status was applied to all migrants arriving from a country with a national policy of universal BCG vaccination [14] and adjusted based on 36-year rates of vaccine coverage [15].

**References**

1. Khan K, Hirji MM, Miniota J, Hu W, Wang J, Gardam M, et al. Domestic impact of tuberculosis screening among new immigrants to Ontario, Canada. Can Med Assoc J. 2015;187(16): E473-81.
2. Canada Facts and Figures: Immigrant Overview Permanent Residents 2014. Government of Canada: Immigration, Refugees and Citizenship Canada. 2015. Available from: http://publications.gc.ca/site/eng/9.512569/publication.html [accessed May 11, 2017]
3. Chin DP, DeRiemer K, Small PM, de Leon AP, Steinhart R, Schecter GF, et al. Differences in contributing factors to tuberculosis incidence in U.S. -born and foreign-born persons. Am J Respir Crit Care Med. 1998;158: 1797-803.
4. Alexander DC, Guthrie JL, Pyskir D, Maki A, Kurepina N, Kreiswirth BN, et al. Mycobacterium tuberculosis in Ontario, Canada: Insights from IS6110 restriction fragment length polymorphism and mycobacterial interspersed repetitive-unit-variable-number tandem-repeat genotyping. J Clin Microbiol. 2009;47: 2651-4.
5. Ferebee SH. Controlled chemoprophylaxis trials in tuberculosis. A general review. Bibl Tuberc. 1970;26: 28-106.
6. Horsburgh CR Jr. Priorities for the treatment of latent tuberculosis infection in the United States. N Engl J Med. 2004;350: 2060-67.
7. Horsburgh CR Jr., O’Donnell M, Chamblee S, Moreland JL, Johnson J, Marsh BJ, et al. Revisiting Rates of Reactivation Tuberculosis: A Population-based Approach. Am J Respir Crit Care Med. 2010;182(3): 420-5.
8. Shea KM, Kammerer JS, Winston CA, Navin TR, Horsburgh CR Jr. Estimated rate of reactivation of latent tuberculosis infection in the United States, overall and by population subgroup. Am J Epidemiol. 2014;179(2): 216-25.
9. Comstock GW, Livesay VT, Woolpert SF. The prognosis of a positive tuberculin reaction in childhood and adolescence. Am J Epidemiol. 1974;99(2): 131-8.
10. Campbell JR, Chen W, Johnston J, Cook V, Elwood K, et al. Latent tuberculosis infection screening in immigrants to low-incidence countries: a meta-analysis. Mol Diagn Ther. 2015;19(2): 107-17.
11. Walter ND, Painter J, Parker M, Lowenthal P, Flood J, Fu Y, et al. Persistent latent tuberculosis reactivation risk in United States immigrants. Am J Respir Crit Care Med. 2014;189: 88-95.
12. Aldridge RW, Zenner D, White PJ, Williamson EJ, Muzyamba MC, Dhavan P, et al. Tuberculosis in migrants moving from high-incidence to low-incidence countries: a population-based cohort study of 519 955 migrants screened before entry to England, Wales, and Northern Ireland. Lancet. 2016;388: 2510-8.
13. TB in British Columbia: Annual Report 2014. Provincial Health Services Authority. 2016. Available from: http://www.bccdc.ca/resource-gallery/Documents/Statistics%20and%20Research/Statistics%20and%20Reports/TB/TB_Annual_Report_2014.pdf [accessed May 11, 2017]
14. BCG World Atlas, 2^nd^ Edition. 2017. Available from: http://www.bcgatlas.org [accessed May 11, 2017]
15. WHO-UNICEF Estimates of BCG Coverage. World Health Organization. 2017. Available from: http://apps.who.int/immunization_monitoring/globalsummary/timeseries/tswucoveragebcg.html [accessed May 11, 2017]
