# Supplementary material for: Cost-effectiveness of post-landing latent tuberculosis infection control strategies in new migrants to Canada
Source: PLoS One. 2017 Oct 30;12(10):e0186778. doi: 10.1371/journal.pone.0186778 (PMC5662173; doi:10.1371/journal.pone.0186778)
Supplement: S2 Text — (DOCX) [file pone.0186778.s002.docx]

**Results of Univariate Sensitivity Analysis**

To perform our univariate sensitivity analysis, nearly all parameters were varied one-way (low and high) in our analysis of the migrant population under post-landing surveillance using two scenarios: our base case, tuberculin skin test (TST) followed by isoniazid if positive (referred to TST/INH) and the most cost-effective alternative, interferon-gamma release assay (IGRA) followed by rifampin, if positive (referred to as IGRA/RIF). The parameters and how they were varied are reported below (**S4 Table**).

The results of the univariate analysis are reported as change in net monetary benefit (NMB) and displayed using tornado diagrams, colour coded for high and low estimates (as the direction of effect changes for some parameters). **S1 Fig** displays change in NMB using quality adjusted life years (QALYs) as the effectiveness parameter.
