# Supplementary material for: Cost-effectiveness of post-landing latent tuberculosis infection control strategies in new migrants to Canada
Source: PLoS One. 2017 Oct 30;12(10):e0186778. doi: 10.1371/journal.pone.0186778 (PMC5662173; doi:10.1371/journal.pone.0186778)
Supplement: S3 Text — (DOCX) [file pone.0186778.s003.docx]

**Results of Probabilistic Sensitivity Analysis**

Using TB cases as the effectiveness parameter yielded similar results to using QALYs when constructing efficiency frontiers. In the population under surveillance, using an IGRA followed by rifampin treatment was the least expensive intervention, which also resulted in the fewest TB cases (**S2 Fig**). In the total migrant population, similar results were found. While remaining in our current TB surveillance system was the least expensive intervention, the only other intervention to fall on the frontier was IGRA screening followed by rifampin treatment, with this intervention being applied to all new migrants furthest along the frontier (**S3 Fig**).

Development of cost-effectiveness acceptability curves in both the total migrant population and population under medical surveillance using TB cases as the effectiveness parameter was not possible, as all interventions demonstrated similar acceptability**. S5 Table** displays the results for each intervention in the total migrant population at select WTP thresholds, while **S6 Table** displays the results for each intervention in the population under medical surveillance.
