# Supplementary material for: Cost-effectiveness of post-landing latent tuberculosis infection control strategies in new migrants to Canada
Source: PLoS One. 2017 Oct 30;12(10):e0186778. doi: 10.1371/journal.pone.0186778 (PMC5662173; doi:10.1371/journal.pone.0186778)
Supplement: S1 Table — (DOCX) [file pone.0186778.s004.docx]

**S1 Table. Model Optimization Targets**

|  |  |  | TB Incidence Category | | | |
| --- | --- | --- | --- | --- | --- | --- |
|  |  |  | <30 cases | 30-99 cases | 100-199 cases | ≥200 cases |
| Number of TB cases in first 2 years | | |  |  |  |  |
|  | Total TB Cases | | 4.28 | 23.62 | 43.22 | 76.27 |
|  | Referred for surveillance and adherent | | 0.92 | 7.20 | 8.38 | 11.76 |
|  | Referred for surveillance and non-adherent | | 0.31 | 2.48 | 7.07 | 9.07 |
|  | Not referred for surveillance | | 3.05 | 13.95 | 27.77 | 55.44 |
| Population Statistics | | |  |  |  |  |
|  | Total Population | | 74,700 | 61,600 | 54,700 | 69,600 |
|  | Referred for surveillance and adherent | | 370 | 1025 | 882 | 1404 |
|  | Referred for surveillance and non-adherent | | 295 | 792 | 595 | 726 |
|  | Not referred for surveillance | | 74,035 | 59,783 | 53,223 | 67,740 |
