# Supplementary material for: Cost-effectiveness of post-landing latent tuberculosis infection control strategies in new migrants to Canada
Source: PLoS One. 2017 Oct 30;12(10):e0186778. doi: 10.1371/journal.pone.0186778 (PMC5662173; doi:10.1371/journal.pone.0186778)
Supplement: S2 Table — (DOCX) [file pone.0186778.s005.docx]

**S2 Table. Final Results of Optimization**

|  |  |  | TB Incidence Category | | | |
| --- | --- | --- | --- | --- | --- | --- |
|  |  |  | <30 cases | 30-99 cases | 100-199 cases | ≥200 cases |
| LTBI Prevalence in Migrants who were not Referred for Surveillance | | | 0.01094 | 0.06198 | 0.13860 | 0.21826 |
|  | | |  |  |  |  |
| Proportion of Migrants Referred for Surveillance That Had Imported TB | | |  |  |  |  |
|  | Adherent with Surveillance | | 0.00192 | 0.00543 | 0.00637 | 0.00544 |
|  | Non-Adherent with Surveillance | | 0.00048 | 0.00153 | 0.00875 | 0.00956 |
| LTBI Prevalence in Migrants Referred for Surveillance | | | 0.0641 | 0.1862 | 0.3659 | 0.3420 |
