# Supplementary material for: Cost-effectiveness of post-landing latent tuberculosis infection control strategies in new migrants to Canada
Source: PLoS One. 2017 Oct 30;12(10):e0186778. doi: 10.1371/journal.pone.0186778 (PMC5662173; doi:10.1371/journal.pone.0186778)
Supplement: S3 Table — (DOCX) [file pone.0186778.s006.docx]

**S3 Table. Interventions Evaluated**

| Strategy | Description |
| --- | --- |
| TST/INH | This is the base case when applied only to the population under medical surveillance. A tuberculin skin test is completed and, if positive via an induration size ≥10mm, nine months of isoniazid is prescribed. |
| TST/RIF | A tuberculin skin test is completed and, if positive via an induration size ≥10mm, four months of rifampin is prescribed. |
| IGRA/INH | An interferon-gamma release assay is performed and, if positive via manufacturer’s definition, nine months of isoniazid is prescribed. |
| IGRA/RIF | An interferon-gamma release assay is performed and, if positive via manufacturer’s definition, four months of rifampin is prescribed. |
| SEQ/INH | A tuberculin skin test is completed. If positive via an induration size ≥10mm, a confirmatory interferon-gamma release assay is given, and, if positive via manufacturer’s definition, nine months of isoniazid is prescribed. |
| SEQ/RIF | A tuberculin skin test is completed. If positive via an induration size ≥10mm, a confirmatory interferon-gamma release assay is given, and, if positive via manufacturer’s definition, four months of rifampin is prescribed. |
