# Supplementary material for: Cost-effectiveness of post-landing latent tuberculosis infection control strategies in new migrants to Canada
Source: PLoS One. 2017 Oct 30;12(10):e0186778. doi: 10.1371/journal.pone.0186778 (PMC5662173; doi:10.1371/journal.pone.0186778)
Supplement: S4 Table — (DOCX) [file pone.0186778.s007.docx]

**S4 Table. Base Estimates and Univariate Analysis Range**

| Parameter | Estimate | Univariate Analysis Range |
| --- | --- | --- |
| *Costs* |  |  |
| Full INH Treatment | $992 | $804, $1179 |
| Full RIF Treatment | $575 | $464, $686 |
| Complete TST | $31 | $24, $38 |
| Incomplete TST | $21 | $17, $25 |
| IGRA | $54 | $31, $62 |
| Tuberculosis | $20,532 | $16,730, $24,334 |
| Adverse Event | $732 | $549, $916 |
| Hospitalization | $6641 | $5305, $9985 |
| Death | $26,933 | $13,079, $40,788 |
| *QALYs* |  |  |
| LTBI | 0.81 | 0.75, 1.0 |
| Healthy | 0.81 | 0.75, 1.0 |
| Disutility due to AE | 0.2 | 0, 0.5 |
| TB | 0.69 | 0.55, 0.75 |
| Hospitalization | 0.5 | 0.3, 0.7 |
| Dead | 0 | - |
| *Screening Parameters* |  |  |
| TST Sensitivity | 0.782 | 0.50, 0.95 |
| TST Specificity (No BCG) | 0.974 | 0.94, 1 |
| TST Specificity (BCG) | 0.602 | 0.35, 0.87 |
| IGRA Sensitivity | 0.889 | 0.81, 0.95 |
| IGRA Specificity | 0.957 | 0.86, 1 |
| IGRA Indeterminate | 0.06 | 0, 0.18 |
| Complete TST | 0.72 | 0.72, 1.0 |
| Complete Medical Evaluation | 0.78 | 0.6, 1.0 |
| *Treatment Parameters* |  |  |
| Initiate | 0.938 | 0.5, 1 |
| Complete INH | 0.616 | 0.5, 0.7 |
| Complete RIF | 0.814 | 0.7, 0.9 |
| Adverse Event INH | 0.060 | 0.04, 0.12 |
| Adverse Event RIF | 0.027 | 0.01, 0.07 |
| Hospitalization \| AE | 0.01 | 0, 0.02 |
| Death INH | 0.00000988 | 0, 0.0001 |
| LTBI Risk Reduction INH | 0.93 | 0.5, 1 |
| LTBI Risk Reduction RIF | 0.8 | 0.5, 1 |
| Partial Risk Reduction INH | 0.346 | 0, 0.69 |
| Partial Risk Reduction RIF | 0 | 0, 0.69 |
| Adverse Event Duration | 7 days | 3, 17 |
| *TB Parameters* |  |  |
| Death from TB | 0.0476 | 0, 0.08 |
| Reactivation Rate | 0.0011 | 0.0009, 0.0013 |
| Extended Therapy | 0.124 | 0, 0.3 |
| Relapse Rate | 0.0359 | 0.0274, 0.0462 |
| *Surveillance Parameters* |  |  |
| Proportion Adherent | 0.605 | 0.7, 0.8 |
| *Model Parameters* |  |  |
| Discount Rate | 0.015 | 0, 0.03 |
| Time Horizon | 10 years | 25 years, 50 years |

INH: Isoniazid; RIF: Rifampin; TST: Tuberculin Skin Test; IGRA: Interferon-Gamma Release Assay; AE: Adverse Event; QALY: Quality Adjusted Life Year; LTBI: Latent Tuberculosis Infection
