# Supplementary material for: Cost-effectiveness of post-landing latent tuberculosis infection control strategies in new migrants to Canada
Source: PLoS One. 2017 Oct 30;12(10):e0186778. doi: 10.1371/journal.pone.0186778 (PMC5662173; doi:10.1371/journal.pone.0186778)
Supplement: S5 Table — (DOCX) [file pone.0186778.s008.docx]

**S5 Table. Probability (%) an Intervention was Cost-Effective Compared to the Base Case in the Total Migrant Population**

| Intervention | WTP: $0 | WTP: $10,000 | WTP: $20,000 | WTP: $30,000 | WTP: $40,000 |
| --- | --- | --- | --- | --- | --- |
| Universal TST/INH | 0 | 0 | 0 | 0 | 0 |
| TST/INH, ≥200 cases | 0 | 0 | 0 | 0 | 0.15 |
| TST/INH, ≥100 cases | 0 | 0 | 0 | 0 | 0 |
| TST/INH, ≥30 cases | 0 | 0 | 0 | 0 | 0 |
| Universal TST/RIF | 0 | 0 | 0 | 0 | 0 |
| TST/RIF, ≥200 cases | 0.05 | 0.10 | 0.30 | 1.15 | 2.45 |
| TST/RIF, ≥100 cases | 0 | 0 | 0 | 0.05 | 0.15 |
| TST/RIF, ≥30 cases | 0 | 0 | 0 | 0 | 0 |
| Universal IGRA/INH | 0 | 0 | 0 | 0 | 0 |
| IGRA/INH, ≥200 cases | 0 | 0 | 0.05 | 0.30 | 1.15 |
| IGRA/INH, ≥100 cases | 0 | 0 | 0 | 0 | 0.20 |
| IGRA/INH, ≥30 cases | 0 | 0 | 0 | 0 | 0 |
| Universal IGRA/RIF | 0 | 0 | 0 | 0.05 | 0.35 |
| IGRA/RIF, ≥200 cases | 0.05 | 0.20 | 1.40 | 4.90 | 10.95 |
| IGRA/RIF, ≥100 cases | 0 | 0.1 | 0.40 | 1.55 | 5.55 |
| IGRA/RIF, ≥30 cases | 0 | 0 | 0.15 | 0.40 | 1.55 |
| Universal SEQ/INH | 0 | 0 | 0 | 0 | 0 |
| SEQ/INH, ≥200 cases | 0 | 0.15 | 0.45 | 1.45 | 3.85 |
| SEQ/INH, ≥100 cases | 0 | 0.05 | 0.05 | 0.15 | 0.40 |
| SEQ/INH, ≥30 cases | 0 | 0 | 0 | 0 | 0 |
| Universal SEQ/RIF | 0 | 0 | 0 | 0.15 | 0.60 |
| SEQ/RIF, ≥200 cases | 0.35 | 1.10 | 3.15 | 7.45 | 12.9 |
| SEQ/RIF, ≥100 cases | 0 | 0.10 | 0.40 | 1.75 | 4.55 |
| SEQ/RIF, ≥30 cases | 0 | 0 | 0.05 | 0.60 | 1.55 |

TST: tuberculin skin test; IGRA: interferon-gamma release assay; SEQ: sequential screening; INH: isoniazid; RIF: rifampin; WTP: willingness-to-pay
