# Supplementary material for: Cost-effectiveness of post-landing latent tuberculosis infection control strategies in new migrants to Canada
Source: PLoS One. 2017 Oct 30;12(10):e0186778. doi: 10.1371/journal.pone.0186778 (PMC5662173; doi:10.1371/journal.pone.0186778)
Supplement: S6 Table — (DOCX) [file pone.0186778.s009.docx]

**S6 Table. Probability (%) an Intervention was Cost-Effective Compared to the Base Case in the Population under Medical Surveillance**

| Intervention | WTP: $0 | WTP: $10,000 | WTP: $20,000 | WTP: $30,000 | WTP: $40,000 |
| --- | --- | --- | --- | --- | --- |
| TST/RIF | 97.75 | 94.80 | 90.10 | 86.75 | 83.35 |
| IGRA/INH | 93.80 | 96.45 | 96.75 | 96.40 | 96.25 |
| IGRA/RIF | 99.70 | 99.40 | 98.85 | 98.35 | 97.6 |
| SEQ/INH | 98.95 | 96.45 | 92.45 | 86.70 | 81.00 |
| SEQ/RIF | 99.35 | 99.00 | 96.50 | 93.75 | 91.15 |

TST: tuberculin skin test; IGRA: interferon-gamma release assay; SEQ: sequential screening; INH: isoniazid; RIF: rifampin; WTP: willingness-to-pay
