# Supplementary material for: Cost-effectiveness of post-landing latent tuberculosis infection control strategies in new migrants to Canada
Source: PLoS One. 2017 Oct 30;12(10):e0186778. doi: 10.1371/journal.pone.0186778 (PMC5662173; doi:10.1371/journal.pone.0186778)
Supplement: S1 Formula — (DOCX) [file pone.0186778.s010.docx]

**S1 Formula.** Chance of Remote Transmission Occurring per Reactivation

$$\left( \frac{1}{Proportion of Cases Due to Reactivation} \right)-1$$
