# Supplementary figures and images for: Cost-effectiveness of post-landing latent tuberculosis infection control strategies in new migrants to Canada
Source: PLoS One. 2017 Oct 30;12(10):e0186778. doi: 10.1371/journal.pone.0186778 (PMC5662173; doi:10.1371/journal.pone.0186778)

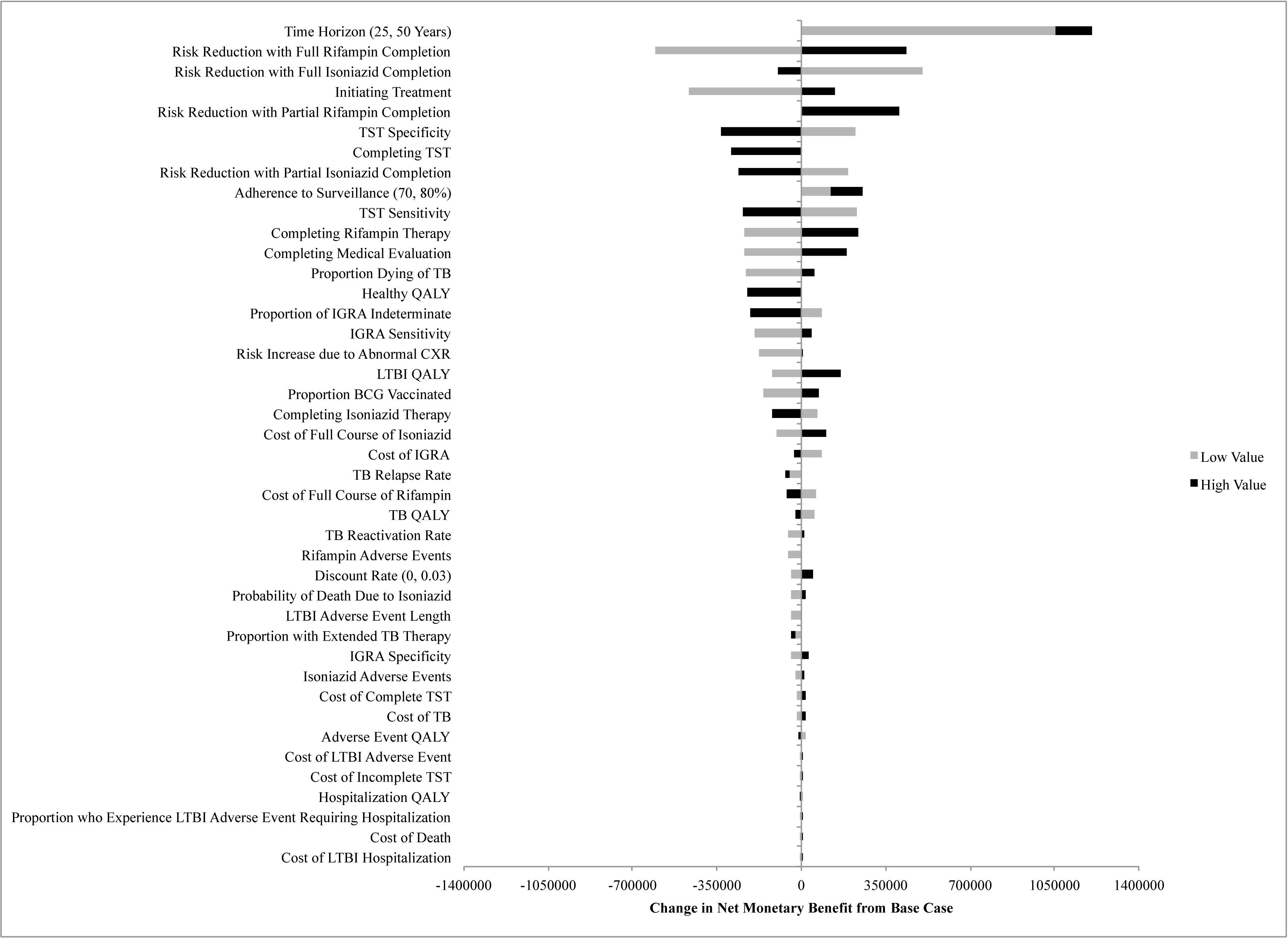

Supplement: S1 Fig — The figure is ordered in the direction of absolute effect (i.e. not in order of direction of effect) on the net monetary benefit in relation to what was calculated in deterministic analysis. QALY: quality adjusted life years; TST: tuberculin skin test; IGRA: interferon-gamma release assay; LTBI: latent tuberculosis infection; TB: tuberculosis; CXR: chest x-ray. (TIF) [file pone.0186778.s011.tif]

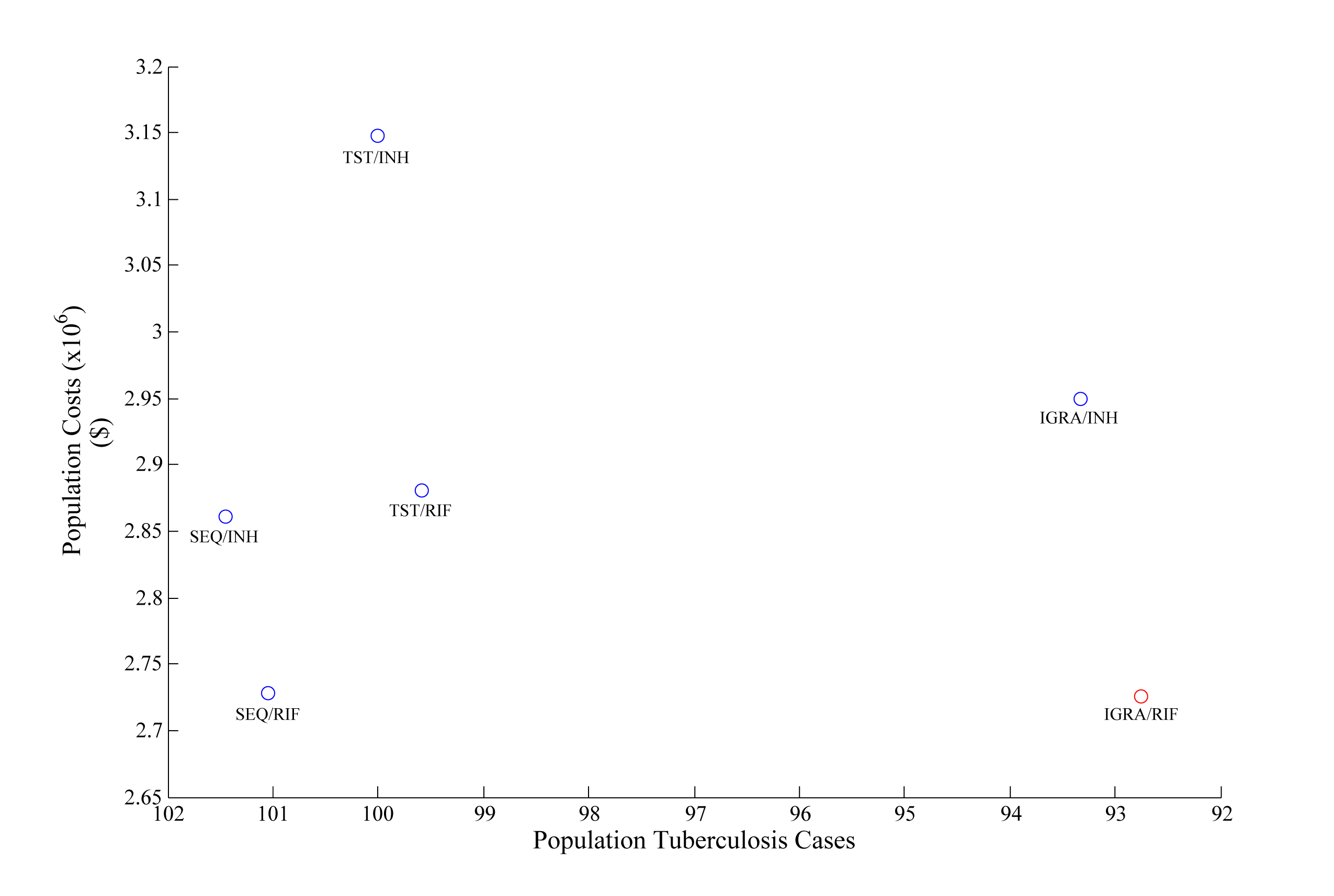

Supplement: S2 Fig — The frontier is read from left to right, with interventions connected if they fall on the frontier; the x-axis is in the reverse direction for ease of understanding. Interventions subsequent to the initial intervention have an increased cost, but an increased benefit (reduction in TB cases), and represent the next best value at increasing funding thresholds. The slope between two connected interventions represents cost-effectiveness: a steeper slope represents poorer cost-effectiveness between interventions, while a shallow slope represents better cost-effectiveness. Intervention(s) that are extendedly dominated have a higher cost-per TB case prevented and more population TB cases than the subsequent intervention on the frontier and are therefore less efficient. SEQ: Sequential Screening; RIF: rifampin therapy; INH: isoniazid therapy; TST: tuberculin skin test; IGRA: interferon-gamma release assay; TB: tuberculosis. (TIF) [file pone.0186778.s012.tif]

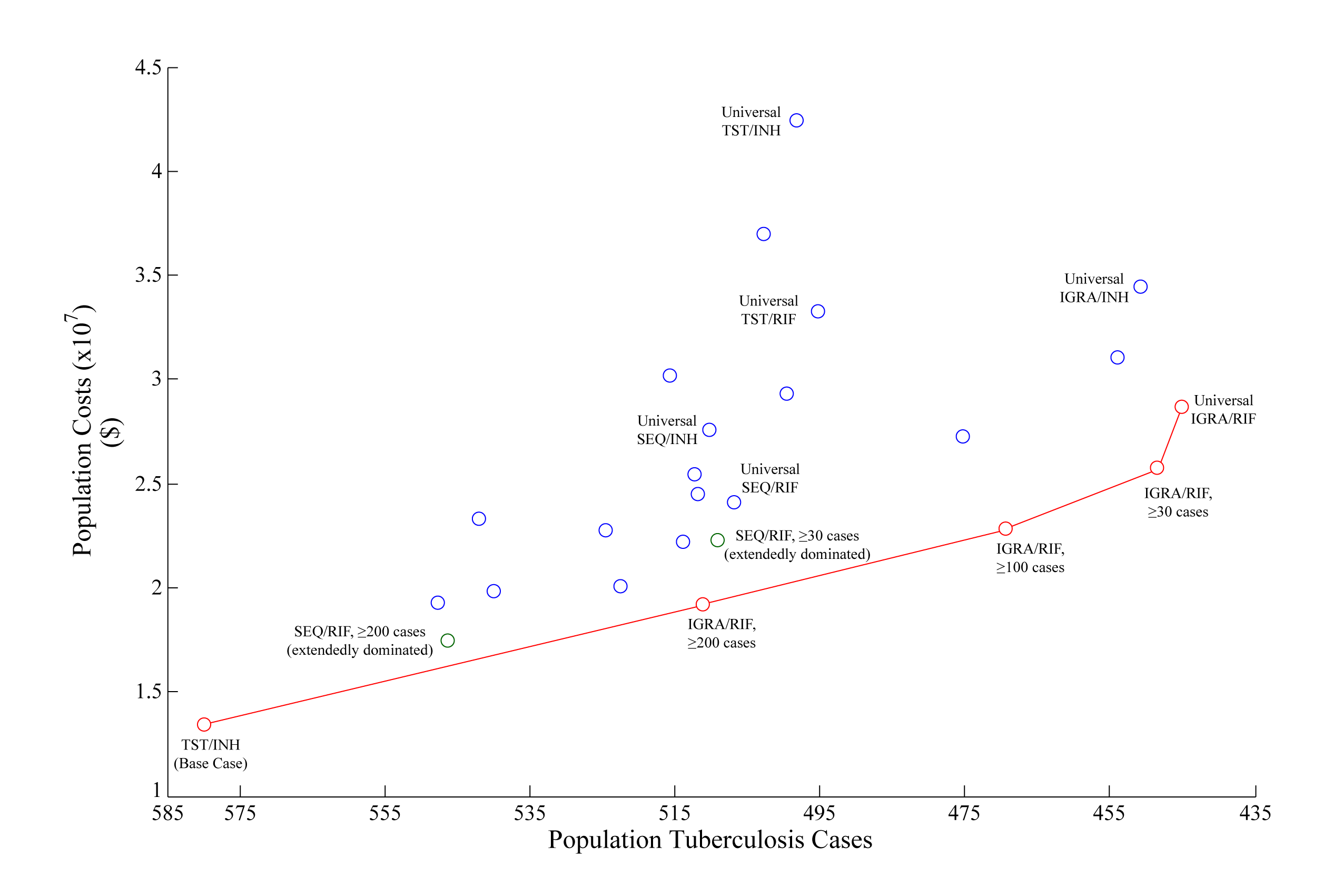

Supplement: S3 Fig — The frontier is read from left to right, with interventions connected if they fall on the frontier; the x-axis is in the reverse direction for ease of understanding. Interventions subsequent to the initial intervention have an increased cost, but an increased benefit (reduction in TB cases), and represent the next best value at increasing funding thresholds. The slope between two connected interventions represents cost-effectiveness: a steeper slope represents poorer cost-effectiveness between interventions, while a shallow slope represents better cost-effectiveness. Intervention(s) that are extendedly dominated have a higher cost-per TB case prevented and more population TB cases than the subsequent intervention on the frontier and are therefore less efficient. SEQ: Sequential Screening; RIF: rifampin therapy; INH: isoniazid therapy; TST: tuberculin skin test; IGRA: interferon-gamma release assay; TB: tuberculosis. (TIF) [file pone.0186778.s013.tif]
